# Fluoride-Controlled Riboswitch-Based Dampening of Gene Expression for Cloning Potent Promoters

**DOI:** 10.3389/fgene.2021.591543

**Published:** 2022-01-21

**Authors:** Vesta Korniakova, Aurélie Devinck, Marie-Christine Groleau, Eric Déziel, Jonathan Perreault

**Affiliations:** INRS - Centre Armand-Frappier Santé Biotechnologie, Boulevard des Prairies, Laval, QC, Canada

**Keywords:** plasmid, promoter, regulatory region, luciferase, translational fusion, fluoride riboswitch, *Burkholderia*, *Pseudomonas*

## Abstract

Bioreporter systems based on detectable enzyme activity, such as that of beta-galactosidase or luciferase, are key in novel bacterial promoter discovery and study. While these systems permit quantification of gene expression, their use is limited by the toxicity of the expressed reporter enzymes in a given host. Indeed, the most potent promoters may be overlooked if their activity causes a lethal overproduction of the reporter genes when screening for transcriptional activity of potential promoter sequences with the *luxCDABE* cassette. To overcome this limitation, a variation of the mini-CTX-*lux* plasmid has been designed which allows reduction of promoter activity *via* the addition of an adjacent fluoride riboswitch. The riboswitch adds a layer of regulation between the promoter and the reporter gene, allowing cloning of stronger promoters by weakening expression, while giving the potential to induce with fluoride to provide a good signal for weaker promoters, thus circumventing limitations associated with reporter toxicity. We noticed the riboswitch potential portability issues between species, suggesting caution when using riboswitches non-native to the species where it is being used. This study introduces a new molecular biology tool which will allow for the identification of previously unverifiable or uncharacterized potent promoters and also provides a cloning vector for translational fusion with luciferase in a plasmid compatible with many species such as from the genera *Burkholderia* and *Pseudomonas*.

## Introduction

Reporter genes encoding for proteins which are easily detectable through sensitive and simple means (colorimetry, fluorescence, luminescence) are key elements to numerous gene expression studies and critical to decipher regulatory elements, including the discovery of new promoters and their characterization in terms of strength and dynamics. Common reporter proteins include β-galactosidase, Green Fluorescent Protein (GFP) and luciferase; detected either by spectrophotometry, fluorimetry or luminometry, respectively. As a rule of thumb, when gene regulatory elements are cloned upstream of a reporter gene, a high reporter protein signal indicates a strong promoter, while a low signal is attributed to a weak promoter. Strategies have been developed to allow for weak promoter detection and characterization *via* reporter gene assays (Guo et al., 2019), however to our knowledge, no strategy for the detection and study of circumstantially lethal potent promoters, have been suggested. Classical gene reporter assays may have biases against the most potent promoters. The toxicity caused by overexpression of reporter proteins could inhibit the growth of potential clones causing an important gap in new promoter discovery.

Many DNA cloning experiments are not successful and are deemed to be technical mysteries. This failure may appear initially as a cloning gap in full genome screens or as an absence of transformed colonies for a given construct in a species of interest other than the shuttle species. A possible reason for these failures may be that an overexpression of the detectable protein in the designed construct has caused a lethal metabolic burden for the cell and thus an absence of viable construct-validated clones. Previously, it has been shown through genome sequencing of clone-based assemblies that many occurring cloning gaps were not technical failures but rather a consequence of the sequences coding for toxic products (Kimelman et al., 2012). As cloning and transformation experiments often involve propagating the construct across different species, constructs must be compatible with the cloning hosts being manipulated in order for an experiment to be successful. While toxicity level thresholds of different reporter genes, their substrates, or byproducts vary depending on the host organism, overexpression of any protein can potentially be toxic (Bolognesi and Lehner, 2018). In fact, toxicity has been previously reported for luciferase substrate N-decyl aldehyde in *Saccharomyces cerevisae* and *Caenorhabditis elegans* (Hollis et al., 2001); for constitutive expression of *Gaussia princeps* luciferase (Gluc) in *Escherichia coli* (Liu et al., 2014); for β-galactosidase expression in *E. coli* under osmotic stress (Malakar et al., 2014); and for GFP in *S. cerevisae* (Kintaka et al., 2016). One study concluded that for a number of proteins, the overexpression burden limit in *S. cerevisae* is achieved for normally non-harmful proteins once it constitutes up to 15% of the total cellular proteins (Eguchi et al., 2018).

**FIGURE 1:**
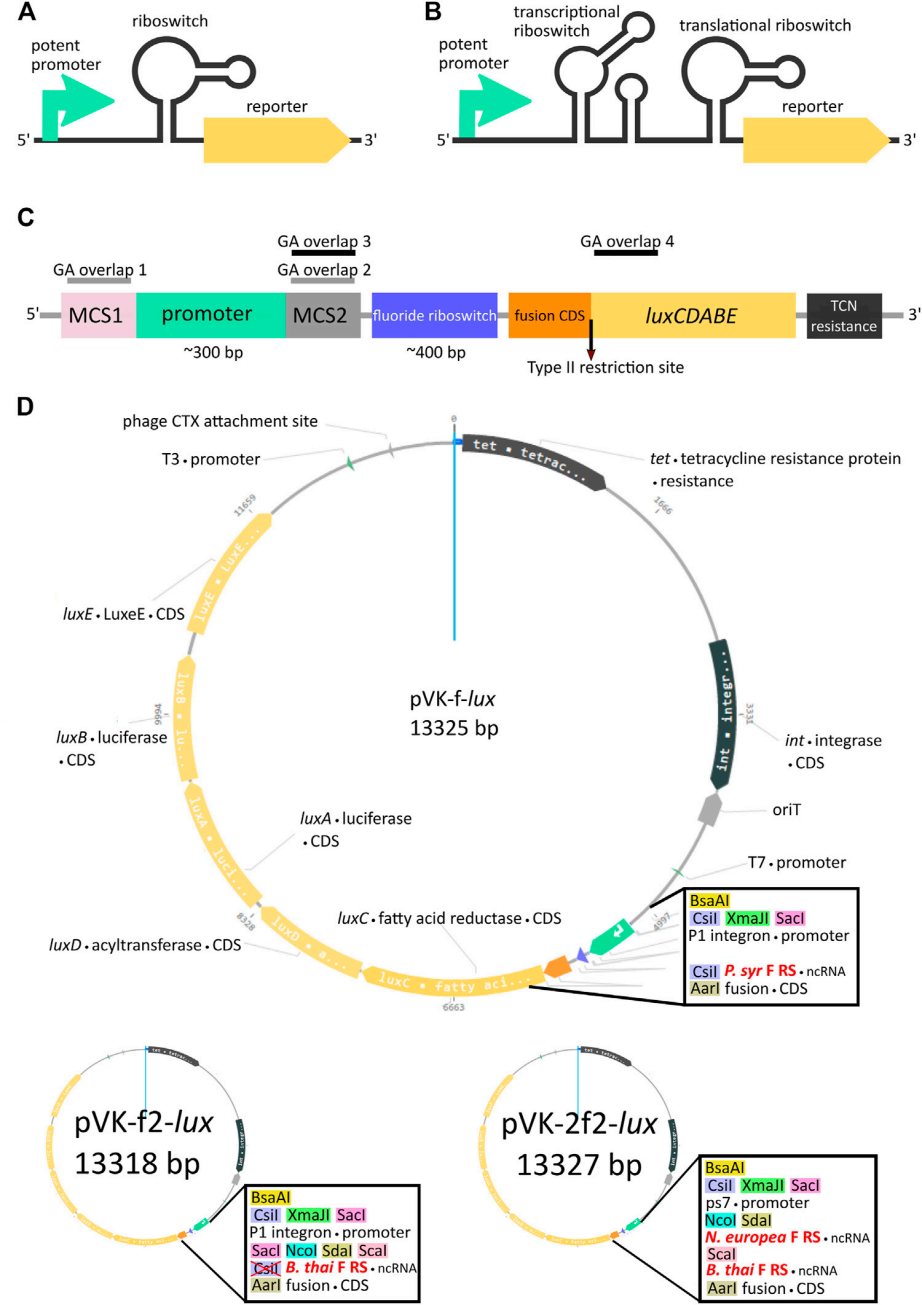
Riboswitch-mediated reporter expression under the control of a potent promoter. Schematic diagram of a potent promoter dampening strategy with one **(A)** or two **(B)** riboswitches. **(C)** Key features of pVK-f-*lux*, a parts-swappable mini-CTX-*lux* derived backbone for convenient cloning of promoters and RNA regulatory parts. MCS1 contains CsiI-XmaJI-SacI restriction sites and MCS2 contains NcoI-SdaI-ScaI restriction sites. A type IIS restriction site is located at the beginning of *luxC* allowing digestion into the second codon for scar-free translational fusion. GA overlaps for double digestions with SacI and NcoI or ScaI and AarI have been designed for allowing interchangeability of parts as described (Supplementary Material: Quick User Manual for pVK-f-*lux*). These double digestions are buffer compatible and yield fragments visible on an agarose gel (approximately 300 and 400 bp in length, see Supplementary Figure S3 for more details). **(D)** Plasmid map of pVK-f-*lux* and pVK-f2-*lux.*

The goal of the cloning strategy described in this paper was to decouple the cloning and transformation process from the evaluation of promoter activity in a host organism. To the best of our knowledge, no such strategy has previously been described. To this end we believe riboswitches, ligand-specific RNA *cis*-acting gene regulatory elements, may be important tools for dampening the strength of potent constitutive or potent uncharacterized inducible promoters. By sandwiching an appropriate riboswitch between the potent promoter and the reporter gene, expression levels may be controlled and adjusted down to viable levels during promoter screening or characterization assays (Figures 1A,B). Many expression vectors are designed for protein induction *via* inducible promoters to avoid toxicity of the protein to be purified during cloning and growth, however to our knowledge no vector exists for controlled promoter read-out *via* inducible 5’ UnTranslated Regions (UTRs). Different approaches may be used to reduce expression levels, such as copy number or forced chromosomal integration, this was reviewed in (Camps, 2010) and (Marschall et al., 2016). Others (Xu et al., 2013) have evaluated copy number of plasmids to optimize fatty acid production in *E. coli* and found, expectedly, that high copy number plasmids incurred higher expression which was deleterious to growth in certain conditions. In principle, copy number could be controlled either by mutating the *ori* or, at least in the case of *ColE1* plasmids, mutating and changing the ratios of RNAII and/or RNAI, which control plasmid replication (Standley et al., 2019). However, in addition to limiting us to *ColE1* plasmids, this has been done such that copy number can be achieved with different mutants, but we are not aware of a system that allows control of copy number in a manner similar to induction systems.

When choosing the right promoter-dampening riboswitch for an experiment it is important to consider its compatibility with the shuttle and final host species. Criteria to consider include regulation range dynamics of the riboswitch (including fold induction and regulation mechanism), and whether or not the trigger ligand is endogenous to the species and what systems exist, if any, to control ligand concentrations inside the cell. For this study, we identified the fluoride riboswitch (F RS) as a potential candidate for mitigating the potency of two promoters to be used in a reporter gene system: the constitutive S7 ribosomal protein gene promoter (P_S7_) from *Paraburkholderia xenovorans* strain LB400 and the P1 integron promoter originally from R388, a trimethoprim-resistance broad-host-range plasmid (Zolg and Hanggi, 1981; DeShazer and Woods, 1996; Massey et al., 2011). These promoters were meant to be cloned upstream of the *lux* cassette in a strategically redesigned version of mini-CTX-*lux* (Becher and Schweizer, 2000), a high copy plasmid in *E. coli* or a single copy chromosomal integration plasmid for *Burkholderia thailandensis* and *Pseudomonas aeruginosa*. The fluoride riboswitch has the advantage of controlling gene expression according to concentrations of fluoride, a non-cellular metabolite. This conserved RNA structure is widespread across bacteria and archaea and is known to upregulate, in the presence of fluoride ions, the expression of proteins which manage its exportation, such as the CrcB proteins and the CLC proteins, fluoride-specific channels which act as fluoride/proton antiporters (Weinberg et al., 2010; Baker et al., 2012; Stockbridge et al., 2012). The atomic resolution structure of this riboswitch, was shown to have a four base pairs pseudoknot and two small pseudoknots of a single base pair, with the ligand, fluoride ions, coordinated to 3 Mg^2+^ ions, themselves coordinated by water and the ribose-phosphate backbone (Ren et al., 2012). This widespread riboswitch regulates numerous genes and uses different expression platforms, sometimes with obvious Rho-independent transcription terminators and sometimes presumably through translation regulation (Weinberg et al., 2010; Baker et al., 2012). Typically, F RS have a *K*_*D*_ ∼ 50 μM, according to *in vitro* assays performed with instances of this riboswitch from four different species, but the concentration added in media that will trigger the riboswitch is much higher (mM range) due to active export of fluoride ions (Baker et al., 2012). Additionally, the fluoride riboswitch from a thermophilic archaeon has been previously used as an alternative strategy to inducible promoters for regulating gene expression in hyperthermophiles (Speed et al., 2018).

Our redesigned plasmid, which we named pVK-f-*lux,* features optimal cloning features for allowing to easily swap promoters and 5′UTRs as needed in order to find the right combination for a particular experiment. Additionally, it is optimized to allow for scar-free translational fusion cloning, a feature not often present in reporter vectors, but essential for studying many *cis-*regulatory RNA elements. Our plasmid is designed for Gibson assembly (GA) cloning but may also be used with a restriction enzyme digestion and ligation approach.

## Methods

### DNA Amplification and Reporter Plasmid Construct Assembly

Oligonucleotides from Integrated DNA Technologies (25 nmoles DNA oligonucleotides and 500 ng of gBlocks^®^ Gene Fragments) were used. DNA parts for GA cloning were amplified using the Q5^®^ High-Fidelity DNA Polymerase (New England Biolabs) using a touchdown-gradient PCR protocol as previously described (Korbie and Mattick, 2008) and appropriate primers and template as specified in Supplementary Table S1. Touchdown annealing cycles (−1.2°C/cycle for 10 cycles) were performed from 71°C down to 60.2°C and were followed by 20 cycles of constant annealing temperatures (with five tubes in a gradient from 55°C to 72°C). Reactions with the most specificity were chosen for further cloning steps. Backbone vectors, as per specific cloning attempts (Supplementary Table S1 and Supplementary Table S2) were linearized using restriction enzymes (Thermo Fisher Scientific) as described. GA cloning was carried out using diluted PCR products, unpurified restriction digestion products and the NEBuilder^®^ HiFi DNA Assembly Master Mix kit (New England Biolabs) according to the manufacturer protocol.

### Bacterial Strains and Construction of Reporter Strains

All strains and clones used in this study are enumerated in Supplementary Table S3. All strains were grown at 37°C on a rotary agitator in liquid Luria Broth (Alpha Biosciences) or on Petri dishes of Luria agar (Alpha Biosciences). GA-cloned plasmid constructs were transformed into either *E. coli* strain DH5*α* or strain SM10λpir as follows. Either 2 µL of the GA reaction or 25 ng of the plasmid of interest was added to 100 µL of thawed chemically competent cells on ice and incubated for 20 min. A thermal shock was performed for 40 s at 42°C followed by a 3-min incubation on ice. 300 µL of Luria broth was added to the mix and cells were incubated at 37°C for 1 h with agitation at 250 rpm. 150 µL of cells were spread on a prewarmed selection plate and incubated overnight at 37°C.

The constructs were integrated into the chromosome of *B. thailandensis* E264 by bi-parental conjugation with *E. coli* SM10λpir as follows. Pellets from 1.5 ml of overnight cultures diluted to 0.5 OD_600_ for both *E. coli* SM10λpir donor strains and for *B. thailandensis* E264 *WT* strain were obtained by centrifugation at 7,000 *g*. Each pellet was resuspended in 25 µL of LB and pooled into a single drop on an antibiotic-free Luria agar dish for overnight incubation at 37°C. The resulting growth was resuspended in 1 ml of liquid Luria Broth using a sterile Q-tip and 100 µL was plated on Luria agar selection plates for *B. thailandensis* E264.

Liquid and solid selection media were supplemented with 15 μg/ml tetracycline for *E. coli* strains; and with 25 μg/ml tetracycline, 50 μg/ml gentamycine and 15 μg/ml polymyxin for *B. thailandensis* E264. When required, FH_4_KO_2_ was added to Luria agar selection plates or to liquid media in concentrations ranging from 0 to 31 mM. Transformed reporter strains were verified for luminescence signal using a microplate reader (Cytation 3; BioTek Instruments, Inc.). Plasmids were extracted from transformed *E. coli* strains using the Presto™ Mini Plasmid Kit (Geneaid) and sequences were confirmed by Sanger sequencing carried out at Genome Quebec (Montreal, Canada).

### Testing Gibson Assembly-Based Cloning in the Plasmid

The designed overhangs of the 5′UTR DNA sequence which excludes the promoter region, were tested for compatibility for GA with a ScaI and AaRI digested backbone, by carrying out GAs with inserts containing the NcoI-SdaI-ScaI left overlap sequence of 17 nts and the AarI/luxC right overlap sequence of 21–24 nts (more details in the cloning flow chart in Supplementary Material).

### *Lux* Reporter Assay

To assess time-course riboswitch regulation dynamics in bacteria, *E. coli* DH5*α*, *E. coli* SM10 and *B. thailandensis* strains transformed with constructs of interest containing the P1 promoter and a 5′UTR translational fusion with the bacterial operon *luxCDABE* were first cultured overnight in LB supplemented with the same antibiotic composition as during transformation. Next, cells were pelleted by centrifugation at 15,000 x *g* for 3 min and washed twice with M9 Minimal Media (M9-MM). Cells were then suspended in fresh M9-MM. M9-MM was prepared by combining 200 ml of sterile M9 salts (64 g/L Na_2_HPO_4_-7H_2_O, 15 g/L KH_2_PO_4_, 2.5 g/L NaCl, 5.0 g/L NH_4_Cl with 2 ml of sterile 1 M MgSO_4_ or MgCl_2_, 20 ml of 20% glucose and 100 µL of sterile 1 M CaCl_2_ in a total volume of 1,000 ml of sterile deionised water. Assays were carried out in 96-well microplates from Greiner Bio-One (Microplate, 96 well, PS F-bottom [chimney well], white, med. binding Ref: 655095). Each well contained a total culture volume of 200 μL of antibiotic supplemented 0.5X M9-MM. Cultures were adjusted to an initial optical density at 600 nm (OD_600_) of approximately 0.06. To seal the plate, an optical film was used before reading. Luminescence and OD_600_ readings were recorded at 20 min intervals with a multi-mode microplate reader (Cytation 3; BioTek Instruments, Inc.) for assay total run times between 30 and 60 h.

### Luciferase Reporter Data Analysis

Average blank values for each time-point were subtracted from each corresponding well sample reading. All readings were cropped to start as soon as the OD_600_ reading hit 0.1 for an individual well. For each assay the number of time points used was adjusted to be the same for all samples unless otherwise stated. For determining the plateau OD_600_ value, the average value of the last 58 OD_600_ readings of a time-course luciferase assay was calculated.

### Fold Induction of Total Luciferase Activity

$$FI\;of\;lumtotal\;=\;\frac{f^{-ligand}\left(n\right)}{f^{+ligand}\left(n\right)}$$

where:

$$f^{-ligand}\left(n\right)=\frac{\sum_{i=m}^{n}\;{\left(\frac{lum}{OD}\right)}^{-ligand}\left(i\right)}{\;};f^{+ligand}\left(n\right)=\frac{\sum_{i=m}^{n}\;{\left(\frac{lum}{OD}\right)}^{+ligand}\left(i\right)}{\;}$$

Where *ligand* is fluoride ions (F-);

(*lum/OD)* ^*-ligand*^(*n*) is the luminescence reading for a culture containing a P1+ F RS-*lux* construct of interest in absence of supplementary fluoride for the time point *n* normalized to its OD at 600 nm.

(*lum/OD*)^+ligand^(*n*) is the luminescence reading for a culture containing a P1+ F RS-*lux* construct of interest in presence of supplementary fluoride for the time point *n* normalized to its OD at 600 nm.

For the calculation of error on fold induction, standard deviation of both triplicates were combined with the following formula. $\frac{\text{σ}FI}{FI}=\sqrt{{\left(\frac{\sigma F0}{av.F0}\right)}^{2}+{\left(\frac{\sigma Fx}{av.F\text{x}}\right)}^{2}}$

Where FI is fold induction; σFI is standard deviation of fold induction; σF0 is standard deviation of triplicate at 0 mM F^−^; av. F0 is average of triplicate at 0 mM F^−^; and similarly for Fx (representing the triplicates of each fluoride concentration tested).

### Peak lum/ OD_600_

The maximum peak luminescence value, in Relative Luminescence Units (RLU), was determined for a time-course reporter assay. This value was divided by the corresponding time-point OD_600_.

### Average Lum/OD_600_

The average RLU value for all time points of each technical triplicate (i.e., for each clone, a single pre-culture divided in three wells for cultures with measurements over ∼ 2 days, or as described in figures and text) was divided by the average OD_600_ value for all time points of each triplicate, respectively. Additional replicate experiments were performed to ensure reproducibility, but were not included in statistics. In cases where reproducibility could not be assessed, it is mentioned in the text.

For the double fluoride riboswitch construct, because the luminescence was close to background, for each assay a restricted window of time was used to calculate FI. This window was selected when the average luminescence of triplicates over 1 hour (i.e., for nine data points) was greater than standard deviation for at least 2 hours in a row (in other words, when luminescence was above background). Such luminescence levels were observed only at concentrations of 31 and 62 mM fluoride and, to use the same time window for both concentrations, we limited ourselves from 22h00–29h20 for *E. coli* DH5α and from 7h00–12h00 for *B. thailandensis.*

## Results and Discussion

### The pVK-F-*Lux* Plasmid Allows for Rapid Mixing and Matching Promoters and 5′UTRs

To study *cis-*regulatory RNA elements, we wanted to devise a luciferase reporter with a potent promoter to provide a strong signal. After multiple cloning attempts, sequencing of the only two clones with inserts of the correct size revealed mutations which would explain the lack of luminescence in these clones (Supplementary Figure S1). In that context, the most likely reason for these failed cloning experiments appeared to be a selective pressure against strong expression of this reporter. To us, this highlighted challenges related to the study of strong promoters and how many strong promoters might have been overlooked in past screening attempts. We thus used a fluoride riboswitch as a way to dampen expression independently of the promoter cloned upstream to design a new reporter tool with unique features.

For our design we chose to include three different single cutter restriction enzyme sites as GA overlap flanking sequences for the promoter region, both for the 5′ end, designated as the Multiple Cloning Site 1 (MCS1), and for the 3′end, designated as the Multiple Cloning Site 2 (MCS2) (Figures 1C,D). Two versions of the plasmid were constructed, containing either the *P. syringae* fluoride riboswitch (*P. syr* F RS), termed pVK-f-*lux*, or the *B. thailandensis* E264 fluoride riboswitch (*B. thai F RS*) (Supplementary Figure S2), termed pVK-f2-*lux* (Figure 1D). For the 3′ end of the 5′ UTR part, AarI, a type IIS restriction site was incorporated. If the inner restriction sites are used to cut the backbone to insert a promoter sequence, then the remainder of the MCS1 and MCS2 sequences on the linearized backbone are sufficient as GA overhangs (GA overlap 1 and GA overlap 2 in Figure 1C) and can be added to the insert of interest. Similarly, by digesting the backbone for a 5′ UTR part insert using the innermost (in relation to the insert position) MCS2 site, and the AarI site, the backbone remainder of the MCS2 site may be used for the 5′ GA overhang (GA overlap 3 in Figure 1C) and the beginning 15–20 nucleotides of *luxC* may be used as the 3′ end GA overhang (GA overlap 4 in Figure 1C). Short inserts (18 and 21 bp) could not be cloned, because a small insert size is already known to be detrimental for Gibson assembly (Roth et al., 2014).

The AarI RE site is positioned to cut the backbone directly after the 2nd nucleotide of the 2nd codon of *luxC*, which allows achieving a scar-free translational fusion (i.e., with no MCS sequence between a regulatory element under study and the start codon) with a choice of the desired start codon. This could be useful given that some known examples of non-AUG start codons are important for translational regulation (Hecht et al., 2017). If scar-free translational fusion is not a priority it is recommended to re-incorporate the AarI site and all depleted restriction sites into the constructs to allow for the flexibility of further cloning by using new plasmid constructs as backbones for new experiments rather than needing to start over from the original backbone, as required for many plasmid assembly methods. If at any point the innermost restriction enzyme recognition site of an MCS, in relation to the insert, is not ideal for a particular cloning strategy, when using a newly assembled backbone, there are still two other restriction sites which may be used at each MCS, thus avoiding, in most cases, the necessity to domesticate any inserts (Supplementary Figure S3). Additional information for using this plasmid is available (Supplementary Material: Quick User Manual for pVK-f-*lux*).

### The Fluoride Riboswitch Dampens Reporter Gene Expression and Facilitates Potent Promoter Cloning

**FIGURE 2:**
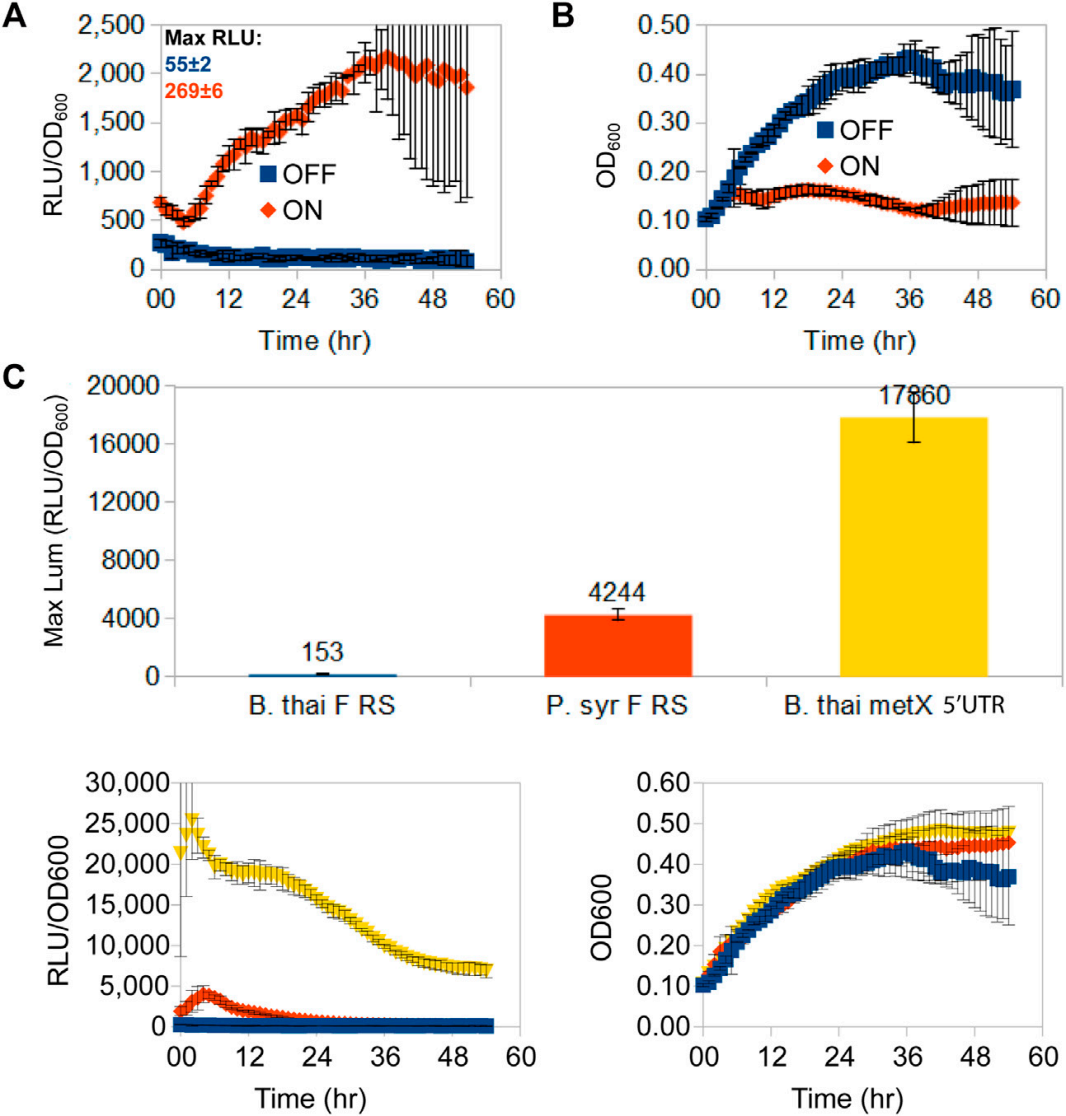
Time-course luminescence induction curves of *B. thailandensis* E264 clones containing the *B. thailandensis* fluoride riboswitch and repression effect. A 54 h time-course *lux* expression assay comparing *lux*/OD **(A)** and growth curves **(B)** of *B. thailandensis* E264 containing the P1-*B. thai* F RS-*lux* (pVK-f2-*lux*) construct*s* (See Supplementary Table S3: *B. thailandensis E264/*P1 *+ B. thai* F) for either an ON-induced state of the fluoride riboswitch in the presence of 31 mM F^−^, or an OFF-repressed state fluoride riboswitch in the absence of F^−^. Cultures were grown and measured on the same 96-well microplate assay run and maximum peak levels of luciferase expression are indicated above curves in Relative Luminescence units (RLU) for un-normalised-to-OD signal strength comparison. The data points represent the means and standard deviations of triplicate values. **(C)** *In vivo* repression capability of three OFF-repressed riboswitch-containing constructs in *B. thailandensis* E264: *B. thailandensis* fluoride riboswitch construct (pVK-f2-*lux—B. thai* F RS), the *P. syringae* fluoride riboswitch (pVK-f-*lux—P. syr* F RS), and the *B. thailandensis metX* 5′UTR (*B. thai metX* 5′UTR). OFF-repression was achieved with 0 mM F- for fluoride riboswitch constructs and with 0.05 mM methionine for the *metX* 5′UTR construct which is suspected to have a regulatory element (Leyn et al., 2014; and unpublished data). RLU per OD_600_ and growth curves are shown as well (bottom left and right respectively). The data points represent the means of triplicate values.

Having chosen the fluoride riboswitch as the candidate regulatory RNA for independent promoter dampening triggered by a non-cellular metabolite, we first needed to evaluate its usefulness to repress elevated expression levels with the option of re-activating expression. For this, we measured luminescence for a fluoride riboswitch reporter construct (pVK-f2-*lux*) in its original host organism (*B. thailandensis* E264) (Figure 2). Depending on the conditions and time, fluoride supplementation causes a ∼ 20X induction of luminescence (RLU/OD_600_) (Figure 2A). The addition of fluoride, or general ion content of media, had no apparent impact on osmotic pressure, since equivalent amounts of chloride (NaCl) made no difference (Supplementary Figure S4). In *B. thailandensis* E264, for OFF conformations (absence of fluoride), the repression capacity at the maximum peak expression of a 54 h time-course luciferase expression curve of the *B. thai* F RS was 28X greater than that of the *P. syr* F RS, and 117X greater than that of the *B. thailandens*is E264 *metX* UTR used as a control (*B. thai metX* 5′UTR; Figure 2C).

Cloning attempts of P_S7_ were unsuccessful with the presence of one fluoride riboswitch, while cloning attempts of the P1 integron promoter were only successful in presence of a riboswitch—either the fluoride riboswitch sequence from *B. thailandensis* E264, the fluoride riboswitch sequence from *P. syringae,* three different 5′UTRs involved in methionine metabolism from *B. thailandensis* E264 or the *yybP-ykoY* riboswitch from *P. aeruginosa* PA14. This suggests that a 5′UTR dampening tool is imperative to repress promoter potency and reduce its toxicity. Cloning the AGGAGC RBS by itself downstream of the P1 promoter was unsuccessful using many GA design strategies (Supplementary Table S1). However, cloning this RBS was successful when integrated within one of the above-mentioned 5′UTR or when in tandem with a weak promoter such as that of the *metK* promoter from *B. thailandensis* E264. Moreover, we have successfully cloned the P_S7_ promoter using a construct comprising two fluoride riboswitches, one from *Nitrosomonas europea* ATCC 19718 and a second from *B. thailandensis* E264, both Betaproteobacteria.

Not all riboswitches enabled successful cloning of the strong P1 promoter, and only a combination of two riboswitches enabled cloning of the P_S7_ promoter, suggesting that the tested riboswitches alone did not sufficiently repress *lux* expression in their OFF conformations. As a reference, one study reported that mRNA coding for the S7 protein was among the top 3% in terms of total RNA quantity, highlighting how strong this promoter is, while *metK* mRNA was in the top 10% (Gorochowski et al., 2019). A full list of successful and unsuccessful cloning experiments enumerated in Table 1 highlights the relation between expression levels and successful cloning of the promoter.

**TABLE 1:** Cloning success of the promoters upstream of the *lux* operon depends on the choice of 5′UTR.

Cloning attempt		Cloning outcome
Promoter	5′ UTR	
P_S7_	5′-AGGAGC-3′ RBS	failed
P _S7_	*B. thailandensis* fluoride riboswitch	failed
P _S7_	*P. syringae* fluoride riboswitch	failed
P _S7_	*B.cereus* and *B.thailandensis* fluoride riboswitch	failed
P _S7_	*N.europea* and *B.thailandensis* fluoride riboswitch	successful
P1 integron	5′-AGGAGC-3′ RBS	failed
P1 integron	5′-AGGAGU-3′ RBS	failed
P1 integron	*E. coli thiM* TPP riboswitch	failed
P1 integron	*B. thailandensis thiC* riboswitch	failed
P1 integron	*B. thailandensis* mini-*ykkC* riboswitch	failed
P1 integron	*B. thailandensis metK* 5′UTR ^a^ ^,^ ^b^	failed
P1 integron	*B. thailandensis* fluoride riboswitch	successful
P1 integron	*P. syringae* fluoride riboswitch	successful
*B. thailandensis metK* promoter	5′-AGGAGC-3′ RBS	successful
P1 integron	*B. thailandensis metX* 5′UTR^b^	successful
P1 integron	*B. thailandensis metZ* 5′UTR ^b^	successful
P1 integron	*B. cereus* fluoride riboswitch + *B. thailandensis* fluoride riboswitch	successful (but not inducible)
P1 integron	*P. aeruginosa* PA14 *yybP-ykoY* riboswitch	successful

a Three different construct designs of varying lengths were attempted (not shown).

b The metK, metX and metZ 5′UTR, from B. thailandensis were suspected to have regulatory elements (Leyn et al., 2014), which we confirmed (unpublished data).

The fluoride riboswitch may be a useful tool for screening for a wide range of promoters and not only those which are potent. For example, a library of unknown sequences containing possible promoters may be cloned into pVK-f-*lux* with an up-regulating fluoride riboswitch in the target host such that the same library of clones can be screened for reporter gene activity in the presence or absence of fluoride. In this way, a screen in the absence of fluoride (with maximum repression), would yield those clones containing the most potent promoters. In parallel, another screen with added fluoride to re-activate riboswitch-mediated repression would allow to detect weaker promoters. Ideally, fluoride threshold tolerance of the target species during transformation and reporter assays as well as the timing of the expression pattern should be determined prior to screening.

**FIGURE 3:**
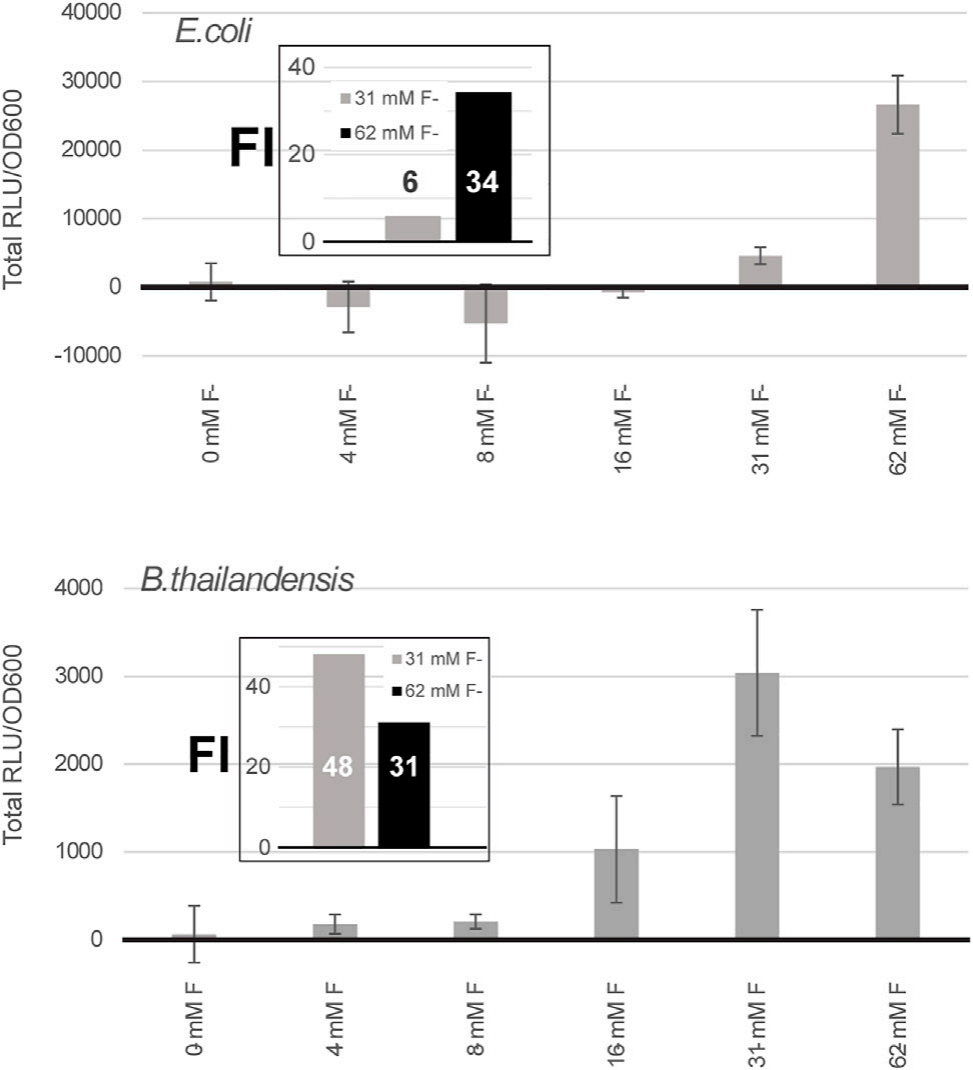
A double fluoride riboswitch construct with P_S7_ is strongly repressed and inducible. Total luciferase activity in cultures was calculated over an incubation period of a few hours, ∼ 7 h for *E. coli* DH5α and 5 h for *B. thailandensis,* these time points were selected because they were above background, as expression remained low even when induced (Supplementary Figure S6). Both were transformed with the *B. thailandensis* E264 fluoride riboswitch construct which has an additional fluoride riboswitch from *Nitrosomonas europea* upstream (pVK-2f2-*lux*). The plasmid is replicative in *E. coli* strains and integrative in *B. thailandensis* E264. Both pre-culture media (LB) and expression media (0.5X M9-MM) were supplemented with appropriate antibiotics depending on the species (see Methods). Fluoride induction concentrations are as indicated. Concentrations were chosen according to the maximum induction effect. The values for each sample represent the means and standard deviations of triplicates on the same microplate.

To test the possibility of using the fluoride riboswitch as a screening tool to mediate promoter potency during transformation of strong promoters (such as P_S7_ or P1 integron promoters), the viability of *E. coli* DH5α and *E. coli* SM10λpir transformant cells was assessed in the presence of 10 and 15 mM fluoride on selection plates. *E. coli* DH5α showed reduced viability at both concentrations as illustrated by the reduced number of visible colonies (Supplementary Figure S5). Colonies which grew on fluoride supplemented plates were also visibly smaller for all tested constructs. Sequenced plasmid extractions of overnight inoculations of the P_S7_ promoter + *B. thai* F RS-*lux* in liquid media with corresponding fluoride concentrations revealed non-functional mutants for all cases even those for which *lux* expression should have been repressed in fluoride supplemented media. We suspect that the cloning failure of P_S7_ in this experiment was due to insufficient repression of *lux* expression rather than fluoride concentration levels in the transformation media as sequencing results were similar to previous Gibson assembly attempts in absence of fluoride and using different GA designs for the same construct (Supplementary Material). Overall 33% of sequenced clones of the P_S7_ promoter + *B. thai* F RS-*lux* attempts from Supplementary Figure S5 contained a 56 nt addition and a point mutation of C285T, in reference to the P_S7_ sequence, 37% had a gap, and 30% had an unrelated sequence included as the insert (Supplementary Table S4). The effect of fluoride on growth in liquid expression media was also tested and *B. thailandensis* E264 clones with chromosomally integrated P1+riboswitch-*lux* constructs for the *B. thailandensis* or the *P. syringae* fluoride riboswitches. All were shown to grow equally well in 31.25 and 62.5 mM fluoride supplemented liquid media (as seen in Figure 2), demonstrating their tolerance to fluoride presence. The P_S7_ *N. europea* + *B. thailandensis* fluoride riboswitches construction was tested using different fluoride concentrations in *E. coli* DH5α and in *B. thailandensis* E264. In *E. coli* DH5α we saw an increase in luciferase expression at 62 mM of fluoride with a fold change of 25 compared to the condition without fluoride (Figure 3). This data suggests that with a double fluoride riboswitch construct we are able to clone the strong P_S7_ promoter and analyze its action in relation to luciferase expression. However, in spite of an apparently strong induction, this double-riboswitch construct is limited by a strong repression. Indeed, the ∼ 30 fold induction from Figure 3 hides a very low expression even when driven by P_S7_ (Supplementary Figure S6).

**FIGURE 4:**
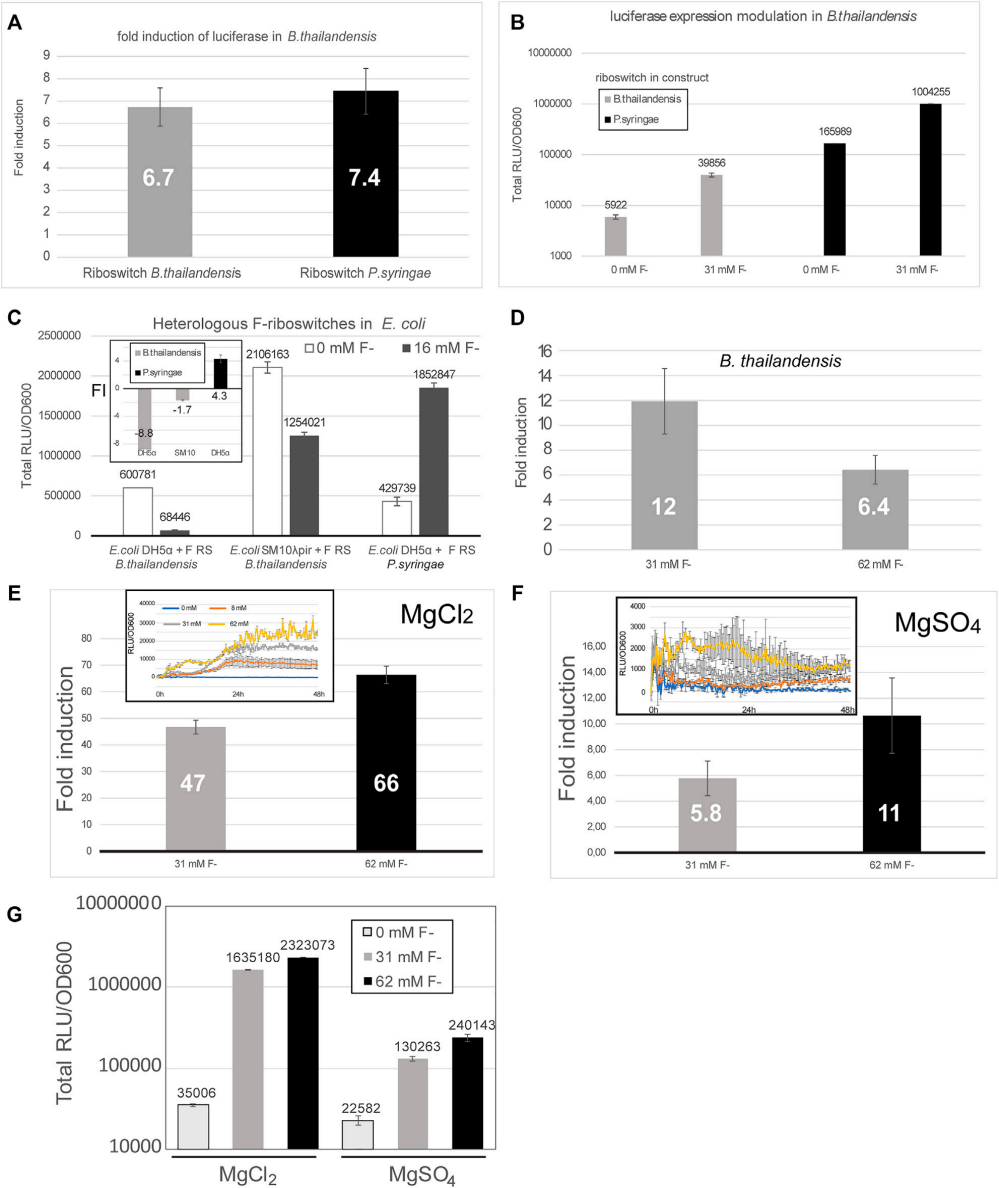
The fluoride riboswitch modulation is affected by media and host strain. **(A)** Fold induction (FI) of total luciferase activity in cultures was calculated over an incubation period of ∼ 40 h (*B. thailandensis* transformed with the *B. thailandensis* E264 fluoride riboswitch construct—pVK-f2-*lux*) and ∼55 h (*B. thailandensis* E264, transformed with *P. syringae* fluoride riboswitch construct—pVK-f-*lux*). Both plasmids are replicative in *E. coli* strains and integrative in *B. thailandensis* E264. Both pre-culture media (LB) and expression media (0.5X M9-MM) were supplemented with appropriate antibiotics depending on the species (see Methods). **(B)** The same results are represented with the direct luminescence (sum) data. **(C)** Fluoride induction concentrations were 16 mM for *E. coli* DH5α and *E. coli* SM10λpir transformants and 31 mM for *B. thailandensis* E264. Concentrations were chosen according to the maximum induction effect. The FI values for each sample represent the means and standard deviations of triplicates on the same microplate. **(D)** Fold induction (FI) of *B. thailandensis* E264 transformed with the *B. thailandensis* E264 fluoride riboswitch for 31 mM and 62 mM of fluoride. **(E–G)** Different culturing conditions (MgCl_2_ vs MgSO_4_) were also evaluated. A much stronger induction by fluoride can be noticed with MgCl_2_.

Luciferase expression was tested in *E. coli* DH5α, *E. coli* SM10λpir and *B. thailandensis* E264, each transformed with a construct containing the fluoride riboswitch originating either from *B. thailandensis* or from *P. syringae* (Figures 4A–C). While riboswitch modulation varied between conditions, for *B. thailandensis* it was coherent with the expected induction mechanism of F RS (Figures 4A,B,D–G), *B. thailandensis* E264 demonstrated an up-regulation, with a 5.1 fold change, up to 25 fold (Figure 2A) with the addition of fluoride, and even ∼ 65 fold in media with MgCl_2_ vs MgSO_4_ (Figure 4E) which we noticed fortuitously. In contrast, *E. coli* DH5α and *E. coli* SM10λpir yielded a very small up-regulation (if any) and we even observed a down-regulation in some assays (Figure 4C and Supplementary Figure S7A). In other words, we did not obtain reliable results with regards to fluoride-mediated induction for the shuttle vector *E. coli*, for which the riboswitches are not native. Nonetheless, repression apparently still occurred since our ability to clone constructs in *E. coli* closely paralleled the strength of promoters, most likely because of viability issues of constructs that strongly expressed luciferase (Table 1). It should also be noted that expression, and FI, varied considerably depending on media used (0.5X M9 with or without sulfur supplementation, i.e., MgCl_2_ or MgSO_4_) (Figures 4E–G). Moreover, fluoride ions being toxic, a pleiotropic effect is expected, such as reduced growth, especially for *E. coli* in presence of 62 mM fluoride, but also with regards to expression. Indeed, a slight fluoride-dependent repression was observed in *B. thailandensis* transformed with the *B. thai metZ* 5′UTR plasmid, even if this UTR harbors no F RS (Supplementary Figure S8). Other riboswitches were tested as well (Table 1), but either did not dampen expression enough to allow cloning or did not provide as good of a modulation (less than two fold). Regulation sensitivity thresholds were also tested for the P1 + *B. thai* F RS (pVK-f2-*lux*, Supplementary Table S2) constructs transformed into *B. thailandensis* E264 and visible regulation effect was achieved at 3.9 mM for *B. thailandensis* E264/P1 *+ B. thai* F (Supplementary Figure S7). Characterization of presented constructs across different strains is important for understanding the limitations of a fluoride riboswitch-mediated *lux* reporter system. To this end, plasmid replication levels and luciferase expression levels were determined in *E. coli* SM10λpir and *E. coli* DH5α. *E. coli* SM10λpir expresses far more luciferase than *E. coli* DH5α at similar fitness levels based on the plateau OD_600_ value, however counterintuitively *E. coli* DH5α produces 1.4X more of pVK-f2-*lux* than the former (Supplementary Table S5).

When running a time-course expression assays, the duration should be optimized to ensure that an expression peak is attained for the given strain and media conditions. Even if addition of 8 mM or 16 mM fluoride induced luciferase expression in most relevant assays, some discrepancies were observed between some clones, both with regards to exact expression quantitation and growth curves. Additionally, peaks were reached at different times in different fluoride concentrations highlighting the importance of a sufficiently long assay run (Supplementary Figure S9). Oscillating expression (with ups and downs) may also be observed when using the fluoride riboswitch in reporter assays as fluoride concentrations inside bacteria will vary according to the activity of the fluoride export pumps (Supplementary Figure S9).

Characterization is not only important for understanding how different conditions affect riboswitch dynamics but also how they may affect the *lux* cassette enzymes, as the system is composed of five different enzymes (*luxCDABE*). We did test the system’s sensitivity to unrelated inducers/repressors. Effect of chloride (up to 15.6 mM) was tested on *E. coli* clones carrying pVK-f2-*lux,* however no regulatory effect was observed (Supplementary Figure S4). Methionine addition (up to 125 mM) to *B. thailandensis* E264 clones carrying pVK-f2-*lux* also did not have a regulatory effect.

## Conclusion

In this study we designed a plasmid which allows for straightforward swapping of promoters and 5′ UTR translationally fused sequences directly from PCR amplified inserts using Gibson assembly-based homologous cloning. We also included the fluoride riboswitch as a tool for modulating reporter gene expression under the control of strong constitutive promoters, such as the P1 integron promoter, in order to circumvent possible reporter overexpression toxicity in both shuttle and final host species, even if it still has limitations, as exemplified by the cloning of the particularly strong P_S7_ promoter which required the combined repression of two riboswitches. We also illustrate that riboswitches used as cloning tools need to be characterized across shuttle species as well as the target species to ensure optimal use. Indeed, we discovered that in its native species the *B. thailandensis* fluoride riboswitch upregulates expression when supplemented with fluoride, yet this gene induction does not translate well to *E. coli*. This is not the first time such a phenomenon has been observed, there are several accounts of riboswitches discovered in metagenomes, or in bacteria difficult to transform, that do not modulate gene expression in model organisms like *E. coli* (personal communication, Ronald Breaker). Several reasons may explain this phenomenon: the difference in riboswitch expression platform folding kinetics due to difference in RNA polymerase activity across species or the wide gap in the GC% of their respective genomes (67 *vs* 50% for *B. thailandensis* and *E. coli*, respectively). Nevertheless, the repression (even if not necessarily relieved by fluoride) permitted cloning both in *E. coli* and *B. thailandendis*. This work may also serve as an example of riboswitch use to improve current cloning tools. Other riboswitches whose ligands are independent of the host organism’s metabolism and have less pleiotropic effects than fluoride, such as the theophylline synthetic riboswitch (Topp et al., 2010), may provide alternatives to apply the same approach, potentially circumventing some of the project-specific limitations that can be encountered the same way different resistance markers can be more or less appropriate for a given cloning experiment.

## Data Availability

The raw data supporting the conclusions of this article will be made available by the authors, without undue reservation.
