# Supplementary material for: Fluoride-Controlled Riboswitch-Based Dampening of Gene Expression for Cloning Potent Promoters
Source: Front Genet. 2022 Jan 21;12:591543. doi: 10.3389/fgene.2021.591543 (PMC8814607; doi:10.3389/fgene.2021.591543)
Supplement: Supplementary file 1 [file DataSheet1.PDF]

Supplementary Material

for the manuscript

Fluoride-controlled riboswitch-based dampening of gene expression for cloning potent promoters

Vesta Korniakova[1], Aurélie Devinck[1], Marie-Christine Groleau[1], Eric Déziel[1] and Jonathan Perreault[1]

[1] INRS - Centre Armand-Frappier Santé Biotechnologie, 531 boulevard des Prairies, Laval, QC, H7V 1B7, Canada,

To whom correspondence should be addressed:

Phone: 450-687-5010 x4411, fax: 450-686-5301, e-mail: jonathan.perreault@iaf.inrs.ca

Key words: plasmid, reporter, promoter, regulatory region, fluoride riboswitch, *Burkholderia*, *Pseudomonas*, *E. coli*, luciferase, toxicity, cloning, induction, repression, translation fusion

Table of contents

Table S1. List of oligonucleotides used in this study. 4

Table S2. Plasmids used in this study. 10

Table S3. Strains used in this study. 12

Figure S1: Inactive mutant transformants of the P_{S7} ribosomal promoter-*lux* construct may indicate *lux* overexpression lethality in *E. coli* DH5α. 15

Figure S2: Secondary structures of fluoride riboswitches used in this study. 17

Figure S3: Sequence of the optimized cloning area of pVK-f-*lux*. 18

Figure S4. Testing chloride to look for potential osmolarity effects. 20

Figure S5: Transformation of heat-shocked *E. coli* is possible in high fluoride concentrations. ... 21

Table S4. P_{S7} promoter cloning sequencing results 22

Figure S6. Double riboswitch constructs full time course……………………………………………23

Figure S7: Regulation thresholds of the *B. thailandensis* fluoride riboswitch in *B. thailandensis* E264 and in *E. coli* SM10λpir. 25

Figure S8: Fluoride has little effect on expression with the *metZ* 5′UTR in *B. thailandensis*……26

Table S5. pVK-f2-*lux* (P1 + *B. thai* F RS-*lux*) plasmid production and *lux* expression specifications in *E. coli* DH5α and *E. coli* SM10λpir. 27

Quantifying plasmid production in *E. coli* strains DH5*α* and SM10λ*pir* 27

Potent promoter cloning bottleneck in *lux* hints at reporter gene toxicity 28

Figure S9: *P. syringae* fluoride riboswitch activity in *E. coli* DH5α reveals oscillating *lux* expression and variability across transformant strains. 29

Quick User Manual for pVK-f-*lux* 30

References 34

Table S1. List of oligonucleotides used in this study.

Part name	Oligonucleotide name	Sequence 5'-3'[1,2]	PCR oligonucleotides and template	Backbone used for cloning this part and other necessary parts[3]
AaRI restriction site deletion in mini-CTX-lux				
luxC AarI deletion	VK45_F	AACGAAGCAGGTGAGGAGCCCACCTGC**G**TGACTAAAAAAATTTCATTCATTATTAACGG	PCR (170322 3. luxC tube 3): VK45_F/VK77_R/(mini-CTX-*lux*)	mini-CTX-*lux* digested with XcmI/SwaI (with SwaI fragment and P_{S7} + RBS strategy 1) or mini-CTX-*lux* digested with BsaAI/SwaI (with SwaI fragment and P_{S7} + RBS strategy 2)
	VK77_R	CGACGTGATGAAGGTACACACATCTGCCAAGTGGTTGATTAAATTCCACACC**C**GCATTTG		
SwaI fragment	VK43_F	GGCAGATGTGTGTACCTTCATCACG	PCR (170323 SwaI): VK43_F/VK76_R/(mini-CTX-*lux*)	mini-CTX-*lux* digested with XcmI/SwaI (with *luxC* AarI deletion and P_{S7} + RBS strategy 1) or mini-CTX-*lux* digested with BsaAI/SwaI (*luxC* AarI deletion and P_{S7} + RBS strategy 2)
	VK76_R	CACCTTTTAATGCTAACGCATCTCGATATTTAAATGATGAC		
Backbone for P1 + 5'-AGGAGT-3' RBS				
VK145	VK145	AAAGTTACAGGCCAGGAACCACGTACCATGGCCTGCAGGAGTACTGCGAATCCTCCGTCGATCCGGGCGGCCGGCGCGATGAGAACAGGGTTGAAGGACTGCGGAAGGAGCAGCACGATGGACGCTCTGTGACAATTCGAGCTCTGGAAGCTGGCTACGTGAACTTCAACCGTAACCACAAC	gBlock	Ppu21I digested 180222-3-3 (P1.2)
5' non transcribed + 5'UTR regions				

P_{S7} + RBS v1	VK47_F	GAAATTACCCCCATTAAATGGATGGCAAATACAGGCTTATGTCTATACAGCTAGCC	PCR (170322 XcmIpMLS7): VK47_F/VK48_R/(pMLS7)	mini-CTX-*lux* digested with XcmI/SwaI (with *luxC* AarI deletion and SwaI fragment)
	VK48_R	CA**C**GCAGGTGGGCTCCTCACCTGCTTCGTTTCTTCCTTTAACTTTTCAGTTGGAGC		
P_{S7} + RBS v2	VK068_F	CATAATTATGACGAAAGTTACAGGCCAGGAACCACGTAGTCAGAATCTG	PCR (1700203 Bs/pM): VK68_F/VK48_R/(P_{S7} + RBS strategy 1)	mini-CTX-*lux* digested with BsaAI/SwaI (with *luxC* AarI deletion and SwaI fragment)
P1 + 5’-AGGAGT-3’ RBS v1	VK110_F	AAAGTTACAGGCCAGGAACCAC	PCR (171201 5 D.): VK110_F/VK144_R/(VK112)	G1C7 digested with BsaAI/AarI
	VK144_R	CCGTTAATAATGAATGAAATTTTTTTAGTCATGCAGGTGAGTACTCCTGCAGGC		
P1 + 5’-AGGAGT-3’ RBS v2	VK162_F	CCATGGCCTGCAGGAGTACTCACCTGCATGACTAAAAAAATTTCATTCATTATTAACGG	VK162_F + VK163_R	08143-6 digested with ScaI/AarI
	VK163_R	CCGTTAATAATGAATGAAATTTTTTTAGTCATGCAGGTGAGTACTCCTGCAGGCCATGG		
P1 + *B.thai* F RS	VK112	AAAGTTACAGGCCAGGAACCACGTAGTCAGAATCTGATTTTCTATATATTTGTTATTTACATCGTCATAACACAAAAATATAAGAAGCAAGTGTTGGTACGACCAGTTCGCAAGATAGTTAAACAGCAACTTAAGTTGAAATTACCCCCATTAAATGGATGGCAAATACCAGGTCCTAGGGAGCTCGAATTCACGAACCCAGTTGACATAAGCCTGTTCGGTTCGTAAACTGTAATGCAAGTAGCGTATGCGCTCACGCAACTGGTCCAGAACCTTGACCGAACGCAGCGGTGGTAACGGCGCAGTGGCGGTTTTCATGGCTTGTTATGACTGTTTTTTTGTACAGTCTATGCCTCGGGCATCCAAGCAGCAAGCGCGTTACGCCGTGGGTCGATGTTTGATGTTATGGAGCAGCAACGATGTTACGCAGCAGGGCAGTCGCCCTAAAACAAAGTTAGGCAGCCGTTGTGCTGGTGCTTTCTGATAGTTGTTGTGGGGTAGGCAGTCAGAGCTCGATTTGCTTGTCGCCATAATAGATTCACAAGAAGGATTCGACCCGGGCCATGGCCTGCAGGAGTACTCGCCCGGCATCGCGTACAATCCGCGGCTACCGGAGATGGCATGCCTCCGTACAACCGCCGGCGAGCCGGCTGATGATGCCTACGCGTTCCTGGGTGCAGGAGGTCGTAGGCCATCCGT	gBlock	G1C7 digested with BsaAI/AarI

		GACAAGCGGCGTCCTGCCGCCCAGGTTTGATGTCCTGTCGAATCTGGAAATTCATGAAACGTCTGCACACTCTCGAGCCCGTCGCGGCGCTCGCGCATCTGTGCCGCTGGCTCGCGCTGTCGGCCGTCGTCGGCGTGCTCGCCGGCTCGGCATCCGCGCTCTTTCTGCGGGCGCTCGATCTCGCCACCGGCACGCGCGTCGCGCATCCGTGGCTGCTGTGGCTGCTGCCCGCAGCGGGCTTCACCTGCATGACTAAAAAAATTTCATTCATTATTAACGG		
B. thai metK promoter + 5’-AGGAGC-3’ RBS[4]	VK149_F	CGCTCTGTGACAATTCGAGAAAGCCGCTATAATACGGGCTTCCTCTGGAAGCTGGCTACG	VK149_F +VK150_R	P1.0 (pVK145) digested with Eco53KI
	VK150_R	CGTAGCCAGCTTCCAGAGGAAGCCCGTATTATAGCGGCTTTCTCGAATTGTCACAGAGCG		
metK IGR—from *B. thai*	VK126_F	AAAGTTACAGGCCAGGAACCACGTACCATGGCCTGCAGGAGTACTGCGAATCCTCCGTC	(PCR: 20171023 PCR tube 2): VK126_F/VK109_R /(gDNA *B.thai* E264)	G1C7 digested with BsaAI/AarI
	VK109_R	CTGGCCGTTAATAATGAATGAAATTTTTTTAGTATAATCGTTTGCCACGTTC		
5’ non transcribed region				
P1 integron promoter	VK164_p4.6_F	CCCATTAAATGGATGGCAAATACCAGGTCCTAGGGAGCTCGAATTCACGAACCCAGTTG	PCR (180724 P4.6 A): VK164_p4.6_F/VK165_p4.6_R/((VK112 gBlock)	08143-6 digested with CsiI/NcoI
	VK165_p4.6_R	AGTACTCCTGCAGGCCATGGCCCGGGTCGAATCCTTC		
P1 integron promoter—no additional RBS	VK110_F	AAAGTTACAGGCCAGGAACCAC	PCR (180724 P0.2 C): VK110_F/VK175_R /(VK112 gBlock)	G1C7 digested with BsaAI/AarI
	VK175_R	CCGTTAATAATGAATGAAATTTTTTTAGTCATGCAGGTGAGTACTGCAGCTGCCATGGCCCGGG		
P_{S7} promoter	VK158_p4.2_F	CCCATTAAATGGATGGCAAATACCAGGTCCTAGGGAGCTCACAGGCTTATGTCTATACAG	PCR (180724 P4.2 A): VK158_p4.2_F/VK159_p4.2_R/(plasmid pMLS7)	08143-6 digested with CsiI/NcoI or P4.1_1 digested with CsiI/NcoI
	VK159_p4.2_R	AGTACTCCTGCAGGCCATGGGTTTCTTCCTTTAACTTTTCAGTTG		
5’ UTR				

P. syr F RS	VK151 (P4.1)	CCATGGCCTGCAGGAGTACTTTTGGACAGACCTAGCTAAGATCGGCGCATTGGAGATGGCATTCCTCCATTAACAAACCGCTGCGCCCGTAGCAGCTGATGATGCCTACAGAAACCTGATCAAACCAGGTCTGTAGGCGTTCGCGCTTAGAATCCCTTCTTTGGTCAGGCCCACTTATTTTTTGTGGCTGGCCAAATGTCTAAATTTCGACGACCTGAACAACTCGACTTACTGCCCTATATAGCGAAATGGCTTGCGCTTGCTGGTCTTGTAGCTCTTTTGGCAGGCTCTGCTTCTGCGTTATTCCTGCTTTCTTTGGATCATGCCACCCAGTGGCGAGAAACCCATCCCTGGGTAATCTGGCTCCTGCCAGTGGCCGGCTTTGTCACCTGCATGACTAAAAAAATTTCATTCATTATTAACGG	gBlock	08143-6 digested with ScaI/AarI
metX 5'UTR—from *B. thai*	VK169_F<br>VK171_R	CCATGGCCTGCAGGAGTACTCGGCGATGCTCGGAAAG<br>CCGTTAATAATGAATGAAATTTTTTTAGTCATGCCGATCGATTCCATTCG	PCR (180724 P2.2 H): VK169_F/VK171_R /(gDNA *B.thai* E264)	08143-6 digested with ScaI/AarI
E.coli thiM TPP riboswitch	VK194_F<br>VK209_R	CCATGGCCTGCAGGAGTACTCCTCTGCGATTTATCATCG<br>CCGTTAATAATGAATGAAATTTTTTTAGTGATCAGGTCGACTTGCATAG	PCR (16.190105 P5.1 A): VK194_F/VK209_R /(gDNA *E.coli* K12)	08143-6 digested with ScaI/AarI
B.thai thiC RS	VK196_F<br>VK210_R	CCATGGCCTGCAGGAGTACTATCGTGTGCGCTTGC<br>CCGTTAATAATGAATGAAATTTTTTTAGTGATGGGGTTGGCGTTCATG	PCR (17.190105 P5.2 D): VK196_F/VK210_R /(gDNA *E.coli* K12)	08143-6 digested with ScaI/AarI
B.thai mini-ykkC RS	VK198_F<br>VK211_R	CCATGGCCTGCAGGAGTACTTACTTCCGCTGCCCGGGGACG<br>CCGTTAATAATGAATGAAATTTTTTTAGTGATCCACAGCCAAGGC	PCR (18.190105 P5.3 A): VK198_F/VK211_R /(gDNA *B.thai* E264)	08143-6 digested with ScaI/AarI
P. aeruginosa PA14 *yybP-ykoY* RS	RN_0079_PA14_rs_fw<br>RN_0079.1_PA14_cds_rev	CCATGGCCTGCAGGAGTACTCGCCCCGTCCTAGGGGA<br>CTGGCCGTTAATAATGAATGAAATTTTTTTAGTTTCCGCGAGGGCAACG	PCR:RN_0079_PA14_rs_fw/RN_0079.1_PA14_cds_rev/(gDNA *P.aeruginosa*	08143-6 digested with ScaI/AarI

			PA14)	
B. cereus F RS + *B.thai* F RS	VK180_F	CCATGGCCTGCAGGAGTCTATATGTAATAATTATAG	PCR: VK180_F/VK183_R /(VK187)	08143-6 digested with ScaI
	VK181_R	GTACGCGATGCCGGGCGAGTACTCTCTTTAAATAGCTTGCTC		
	VK187	CTGCAGGAGTACTGCGCCGCGCGCGACCGAAAAGGCGTGATACGATGGGGCCTTGCGTCGATTTGATTTCAGCTTGCGGACGCGGGGCAACCCGAAACAGCTAAAGCGAAGGCCGGCGAGCAGCGCCATGCCGGCCCGAGTCGATAGCTGCTCCGCACACCAAGCCCGCTGATGCCGACGCATGAGCGGGCAAAAAGTTGGTCTGCGCATTCGCCGCGCGGCGCACGGCGGCGATGCGCTTGAAAACGGACACCTGCATGACTAAAAAAATTTCATTCATTATTAACGGGATCCCATGGCCTGCAGGAGTCTATATGTAATAATTATAGGCGATGGAGTTCGCCATAAACGCTGCTTAGCTAATGACTCCTACCAGTATCACTACTGGTAGGAGTCTATTTTTTTGAGCAAGCTATTTAAAGAGAGTACTTTTGGACAGACCTAGCTAAGATC		
B.thai metZ 5'UTR	VK178_F	CGAAAGTTACAGGCCAGGAACCACGTACCATGGCCTGCAGGAGTACTGCGCCGCGCGCGAC	PCR (7.190102 A): VK178_F/VK179_R /(gDNA *B.thai* E264)	G1C7 digested with BsaAI/AarI
	VK179_R	CCGTTAATAATGAATGAAATTTTTTTAGTCATGCAGGTGTCCGTTTTCAAGCGCATCG		
P_{s7} *N.europea*				
Template	AD_TWIST_ P_{s7}	CCATTAAATGGATGGCAAATACCAGGTCCTAGGCTGGCAACCAATTAGCCGGTCAGTAGTTTCAGGTCGCCTGCAAAGGCGGTAATAGCGGTGGTGCCGGAATCGCCGGTGCTGTCGAGTAAGTGGTCACCCGACCAGGCTGGTAAGTCGTTAGGGATGAATCGGGTTAGTTGGTGGCCGCGGGGCTAAAAGTTAGCTCCAACTGAAAAGTTAAAGGAAGAAACCCATGGCCTGCAGGTATAGTGAAGATAAGGAGATGGTGTTCCTCCTTTTGAAGAAACCGCAGCCGTTTAGCGCTGCTGATGACGCCTACAGGACCTGACCTTCGTTAGGGCTGTAGGCGTTCGTGTTTACATCCTTTAGCATTGTCAGCCCGATTGTTTCGAGCGAATGACCAAAATGTCCGATGCAGATAGACACCGACCAAGACGGAGACTAGTACTCGCCCGGCATCGCGTACAATC	Amplify with AD123 and AD124, digest with XmaJI / ScaI	Mini-CTX-lux digest with XmaJI/ ScaI
Amplified template	AD123	CCATTAAATGGATGGCAAATACCAGGT	AD123/124 to amplify twist template	
	AD124	GATTGTACGCGATGCCGGGC		
Sequencing	AD123	CCATTAAATGGATGGCAAATACCAGGT		

	AD112	GGGCTCGAGAGTGTGCAGAC	AD123/112 for sequencing	

[1]Gibson assembly overlaps are underlined.
[2]Mutations in reference to the template are in bold.
[3]Constructs were visualised and designed using Genome Compiler (http://www.genomecompiler.com/).
[4] AGGAGC RBS from the *metK* IGR was taken from CP000086 *Burkholderia thailandensis* E264 chromosome (1204819…204824).

Table S2. Plasmids used in this study.

Plasmid	Description/associated part name	Source	Cloning procedure
mini-CTX-*lux* (Becher and Schweizer, 2000)	Integration vector with a restriction-site-modified *lux* gene cluster (*luxCDABE)* from *Xenorhabdus luminescens*; Tc^R	AF251497	n/a
pMLS7 (Lefebre and Valvano, 2002)	Expression vector with constitutive promoter of the S7 ribosomal protein gene from *Paraburkholderia* sp. strain LB400	Addgene plasmid # 32056	n/a
pG1C7	mini-CTX-*lux* with 56nt insertion to ribosomal promoter S7 from pMLS7, *luxC* ATG:GTG, and AarI site deletion in *luxC*.	This study	GA from numerous parts including the S7 promoter sequence from pMLS7 (contact authors for more details if needed).
pP1.0	Backbone for P1 + 5'-AGGAGT-3' RBS	This study	GA of Ppu21I digested 180222-3-3 (P1.2) and VK145 gblock.
pVK-f2-*lux* (p08143-6)	P1 + *B. thai* F RS	This study	GA of BsaAI and AarI digested G1C7 backbone and and VK112 gblock.
pP1.1	*B. thai metK* promoter + 5'-AGGAGC-3' RBS	This study	GA of Eco53KI digested P1.0 (pVK145) backbone and oligonucleotides VK149_F +VK150_R

p180222-3-3 (pP1.2)	*metK* IGR— from *B. thai*	This study	GA of BsaAI and AarI digested G1C7 backbone and PCR product of VK126_F/VK109_R/(gDNA *B.thai* E264).
pVK-f-*lux* (pP4.1_1)	P1 + *P. syr* F RS	This study	GA of ScaI/AarI digested 08143-6 backbone and VK151 gblock.
pP2.2-2	P1 + *metX* 5'UTR	This study	GA of ScaI/AarI digested 08143-6 backbone and PCR product of VK169_F/VK171_R/ (180724 P2.2 H).
pP5.6	*P. aeruginosa* PA14 yybP-ykoY RS	This study	GA of ScaI/AarI digested 08143-6 backbone and PCR product of RN_0079_PA14_rs_fw/RN_0079.1_PA14/(gDNA *P.aeruginosa* PA14).
pP4.72	*B.cereus* F RS + *B.thai* F RS	This study	GA of ScaI digested 08143-6 backbone and PCR product of VK180_F/VK183_R/(VK187).
pP6.3	*B.thai metZ* 5'UTR	This study	GA of BsaAI and AarI digested G1C7 backbone and PCR product of VK178_F/VK179_R/(gDNA *B.thai* E264).

Table S3. Strains used in this study.

Strain	Lab catalogue	Description	Source
E. coli DH5*α* (Chen et al., 2018)	B1A4	Used for transformation of plasmid constructs	NCBI:txid668369
E. coli SM10λpir (Simon et al., 1983)	B1A5	Used for conjugating plasmid constructs with *Burkholderia* sp. strain E264 and for luminescence assays	BCCM: LMBP 3889
B. thailandensis E264 (Kim et al., 2005)	B1A7	Used for transformation of plasmid constructs	NCBI:txid271848
E. coli DH5*α*/pG1C7	170404GAC7	Obtained from GA transformation test	This study
E. coli DH5*α/*P1 *+ B. thai* F RS	B1B3; p08143-6	Reporter strain used for luminescence assays	This study
E. coli SM10λpir*/*P1 *+ B. thai* F RS	B1B5; p08143-6	Reporter strain used for luminescence assays	This study
B. thailandensis E264/ P1 *+ B. thai* F RS	B1B7; p08143-6	Reporter strain used for luminescence assays	This study
E. coli DH5α*/B. thai metK* promoter + 5'-AGGAGC-3' RBS	B1G5;p P1.1-1	Obtained from GA transformation test	This study
E. coli SM10λpir*/B. thai metK* promoter + 5'-AGGAGC-3' RBS	B3B9; pP1.1-1-1	Obtained from GA transformation test	This study
B. thailandensis E264*/B. thai metK* promoter + 5'-AGGAGC-3' RBS	B3F9;p P1.1-1-1-2	Obtained from GA transformation test	This study
E. coli DH5α*/metK* IGR	B1D3; p180222-3-3/pP1.2	Reporter strain used for luminescence assays	This study
E. coli SM10λpir/metK IGR	B1D6; p180222-3-3/pP1.2	Reporter strain used for luminescence assays	This study

B. thailandensis E264/*metK* IGR	B1E8; p180222-3-3/pP1.2	Reporter strain used for luminescence assays	This study
E. coli DH5α/P1 + *P. syr* F RS strain 1	B1I6; pP4.1-1	Reporter strain used for luminescence assays	This study
E. coli DH5α/P1 + *P. syr* F RS strain 2	B1I7; pP4.1-2	Reporter strain used for luminescence assays	This study
E. coli DH5α/P1 + *P. syr* F RS strain 3	B1I8; pP4.1-3	Reporter strain used for luminescence assays	This study
E. coli SM10λpir/P1 + *P. syr* F RS	B3D5; pP4.1-2-1	Obtained from plasmid transformation test	This study
B. thailandensis E264/P1 + *P. syr* F RS	B3I2; pP4.1-2-1-1	Reporter strain used for luminescence assays	This study
E. coli DH5α/P1 + *metX* 5'UTR	B3E2; pP2.2-2	Reporter strain used for luminescence assays	This study
E. coli SM10λpir/P1 + *metX* 5'UTR	B4A4; pP2.2-2-1	Reporter strain used for luminescence assays	This study
B. thailandensis E264/P1 + *metX* 5'UTR	B4B1; pP2.2-2-2-1	Reporter strain used for luminescence assays	This study
E. coli DH5α/P. aeruginosa PA14 yybP-ykoY RS	B4G4; pP14-1	Obtained from GA transformation test	This study
E. coli SM10λpir/*P. aeruginosa* PA14 yybP-ykoY RS	B5E1; pP5.6-1_1	Obtained from plasmid transformation test	This study
B. thailandensis E264/*P. aeruginosa* PA14 yybP-ykoY RS	B7C1; pP5.6-1_1-1	Obtained from plasmid transformation test	This study
E. coli DH5α/B. cereusr F RS + B.thai F RS	B4E4; pP4.72-1	Obtained from GA transformation test	This study
E. coli DH5α/B.thai metZ 5'UTR	B4C7; pP6.3-1	Obtained from GA transformation test	This study
E. coli SM10λpir/*B.thai metZ* 5'UTR	B5C9; pP6.3-1_1	Obtained from plasmid transformation test	This study

B. thailandensis E264/*B.thai metZ* 5'UTR	B7A9; pP6.3-1_1-1	Obtained from plasmid transformation test	This study

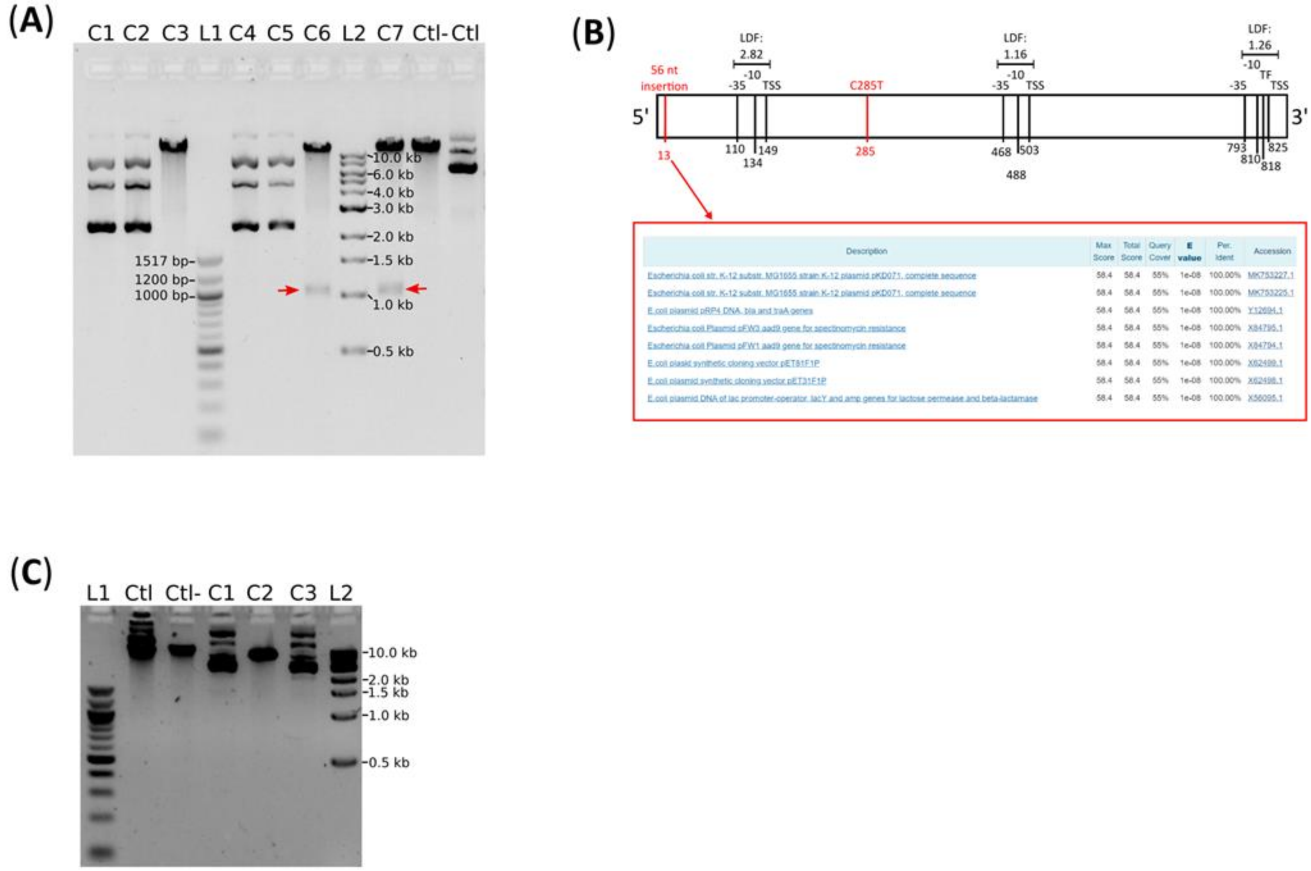

Figure S1: Inactive mutant transformants of the P_{S7} ribosomal promoter-*lux* construct may indicate *lux* overexpression lethality in *E. coli* DH5α.

(**A**)Verification of a cloning and transformation experiment by PstI restriction enzyme digestion of plasmids extracted from *E. coli* clones transformed with a GA reaction of a XcmI/SwaI digested mini-CTX-*lux* backbone and 3 fragments forming a P_{S7} -RBS-*luxC* insert for downstream reporter assays. Ctl- is the original mini-CTX-*lux* plasmid digested with PstI (linearized plasmid, expected size: 12,538 bp) and Ctl is undigested mini-CTX-*lux*. Expected product sizes of a PstI digestion of a successful assembly should yield 12,439 bp and 973 bp products. PCR-amplified assembly fragments (with GA overlaps) were 907 bp (P_{S7} + RBS v1), 1162 bp (*luxC* AarI deletion), and 139 bp (SwaI fragment) and linearized backbone was 11311 bp (**Table S1**). The 7 clones obtained from the experiment are labelled C1-C7. Ladders are designated as L1 (100 bp DNA Ladder from NEB) and L2 (1 kb DNA Ladder from NEB) with indicated size for most relevant bands. Sequencing results were further obtained for C6 and C7 as they revealed expected sized bands (red arrows).

(**B**) Verification of a cloning and transformation experiment by PstI restriction enzyme digestion of plasmids extracted from *E. coli* clones transformed with a Gibson Assembly reaction of a BsaAI/SwaI digested mini-CTX-*lux* backbone and 3 fragments forming a P_{S7} -RBS-*luxC* insert for downstream reporter assays. PCR-amplified assembly fragments were 1057 bp (P_{S7} + RBS v2), 1162 bp (*luxC* AarI deletion), and 139 bp (SwaI fragment) and linearized backbone was 11178 bp (**Table S1**). Expected

product sizes of a PstI digestion of a successful assembly should yield a 12439 bp and a 973 bp product. The 3 clones obtained from the experiment are labelled C1-C3. Ctl- is the original mini-CTX-*lux* plasmid digested with PstI (expected size: 12538 bp) and Ctl is undigested mini-CTX-*lux*. Ladders are designated as L1(100 bp DNA Ladder from NEB), L2(1 kb DNA Ladder from NEB). No sequencing results were further obtained as these results indicate misassembly of fragments.

(**C**) Clones 6 and 7 of the Gibson assembly attempts of the P_{S7}-RBS-*luxCDABE* yielded misassembled constructs for which the former had a point mutation of G334T of the *luxC* sequence (not shown), and the latter had a 56 nt insert in the P_{S7} promoter, as amplified from pMLS7, between C13 and T14, and a point mutation at position 285 (in red). The 850 bp promoter sequence was analysed using BPROM[8] and the elements of the three predicted promoters are noted including the -35 and -10 conserved promoter boxes, the Transcription Start Site position (TSS), and position of the oligonucleotides from the crp Transcriptional Factor binding site (TF) (in black). The Linear discriminant function (LDF) of each predicted promoter is also noted. Nucleotide Blast alignment results of the 56 nt insert are also presented. Nucleotide BLAST search hits of the 56 nt insert sequence against *Escherichia coli* K-12 are presented in the red box.

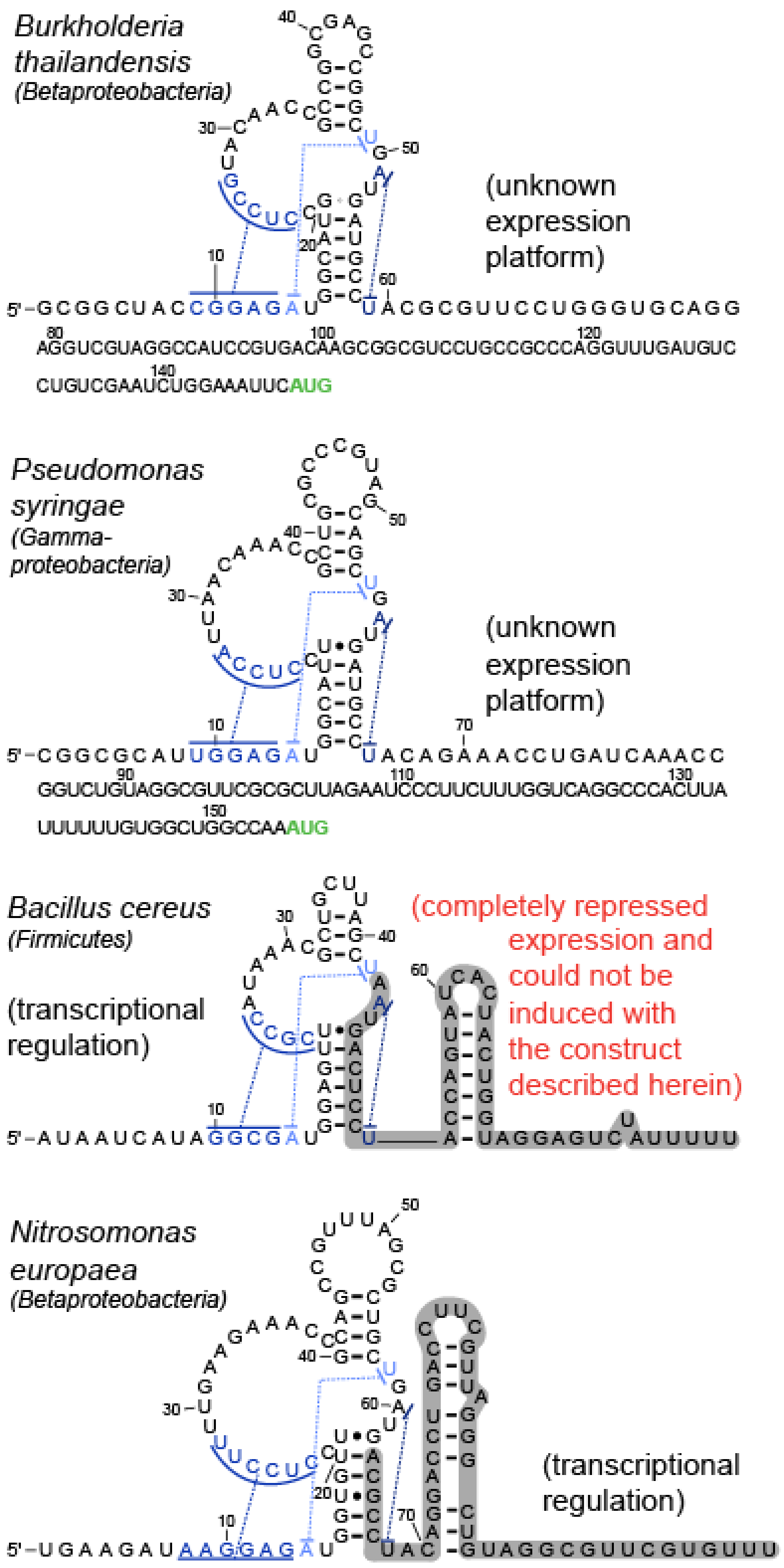

Figure S2: Secondary structures of fluoride riboswitches used in this study.

The secondary structures of the four riboswitches described in this manuscript is pictured. Stems and pseudoknots are as annotated in (Baker et al., 2012 and Ren et al., 2012). The start codon is represented as a green AUG for riboswitches with presumed (but unconfirmed) translational mechanism. Grey shading highlights the portion of sequence presumed to be an expression platform based on a Rho-independent transcriptional terminator.

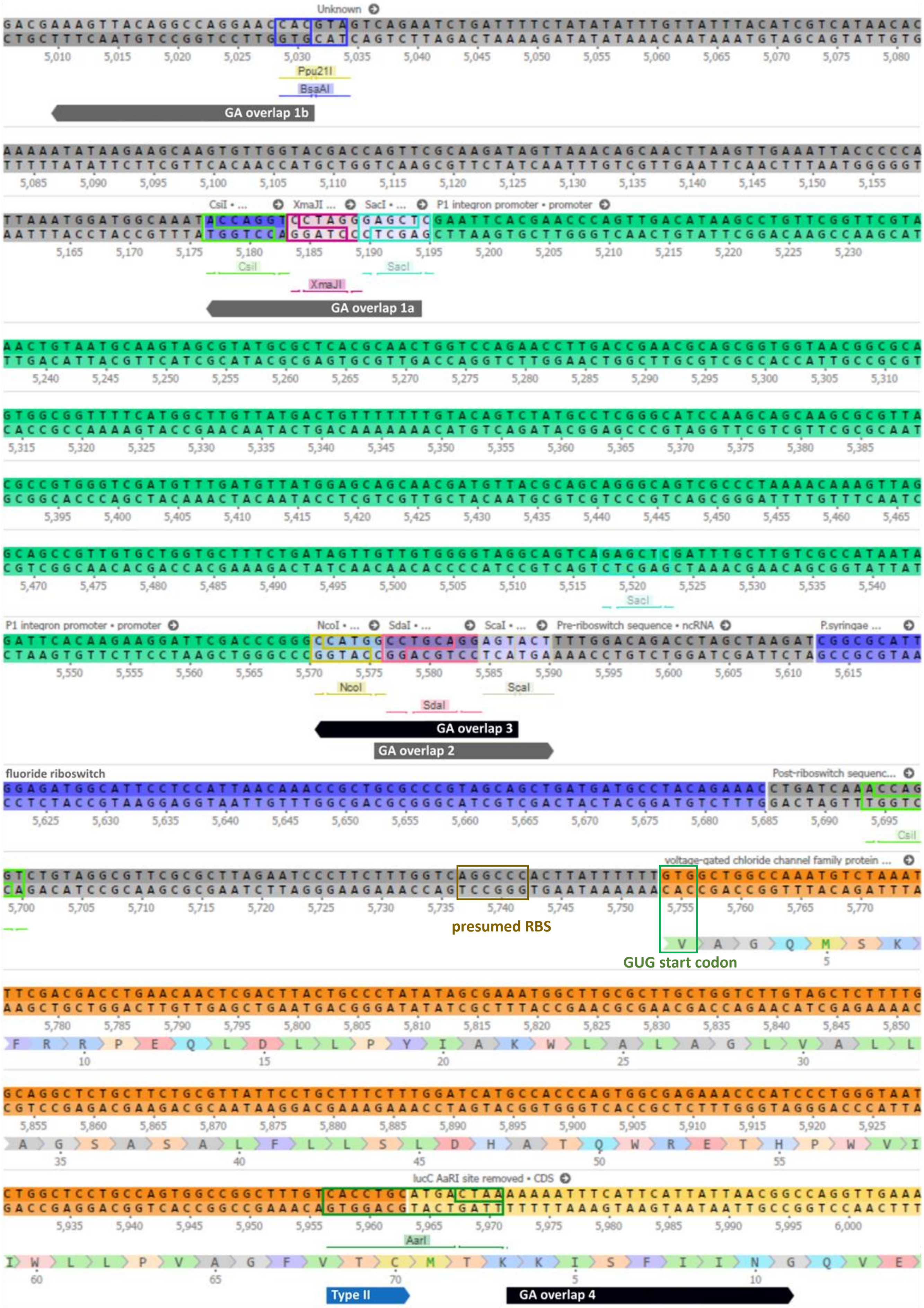

Unknown
Ppu21I
BsaAI
GA overlap 1b
CsiI • ...
XmaJI ...
SacI • ...
P1 integron promoter • promoter
CsiI
SacI
XmaJI
GA overlap 1a
SacI
P1 integron promoter • promoter
NcoI • ...
SdaI • ...
ScaI • ...
Pre-riboswitch sequence • ncRNA
P.syringae ...
NcoI
ScaI
SdaI
GA overlap 3
GA overlap 2
fluoride riboswitch
Post-riboswitch sequenc...
CsiI
voltage-gated chloride channel family protein ...
presumed RBS
GUG start codon
lucC AaRI site removed • CDS
AarI
Type II
GA overlap 4

Figure S3: Sequence of the optimized cloning area of pVK-f-*lux*.

Nucleotide parts are designated by different colours: grey areas are sequences of unknown function, including the pre-riboswitch sequence (5591...5612) and the post-riboswitch sequence (5686...5753) naturally found directly upstream and downstream, respectively, of the fluoride riboswitch in *P. syringae.;* turquoise is the P1 integron promoter (5196...5570); purple is the *P. syringae* fluoride riboswitch (5613…5570); orange is a portion of the gene encoded downstream of the *P. syringae* fluoride riboswitch (5754…5956) with an additional AarI Type IIS recognition site (5957...5963 with blue arrow below sequence); and yellow is the original *luxC* sequence (starting at position 5964). Restriction enzyme recognition sites intended for adding or swapping parts are annotated. GA overlaps for parts design are annotated in dark grey for promoter swapping, using either MCS1 (GA overlap 1a) or an upstream BsaAI RE site (GA overlap 1b) and MCS2 (Gibson overlap 2), and in black for 5'UTR swapping, using MCS2 (GA overlap 3) and AarI RE site (GA overlap 4). Image was put together using Genome Compiler software.

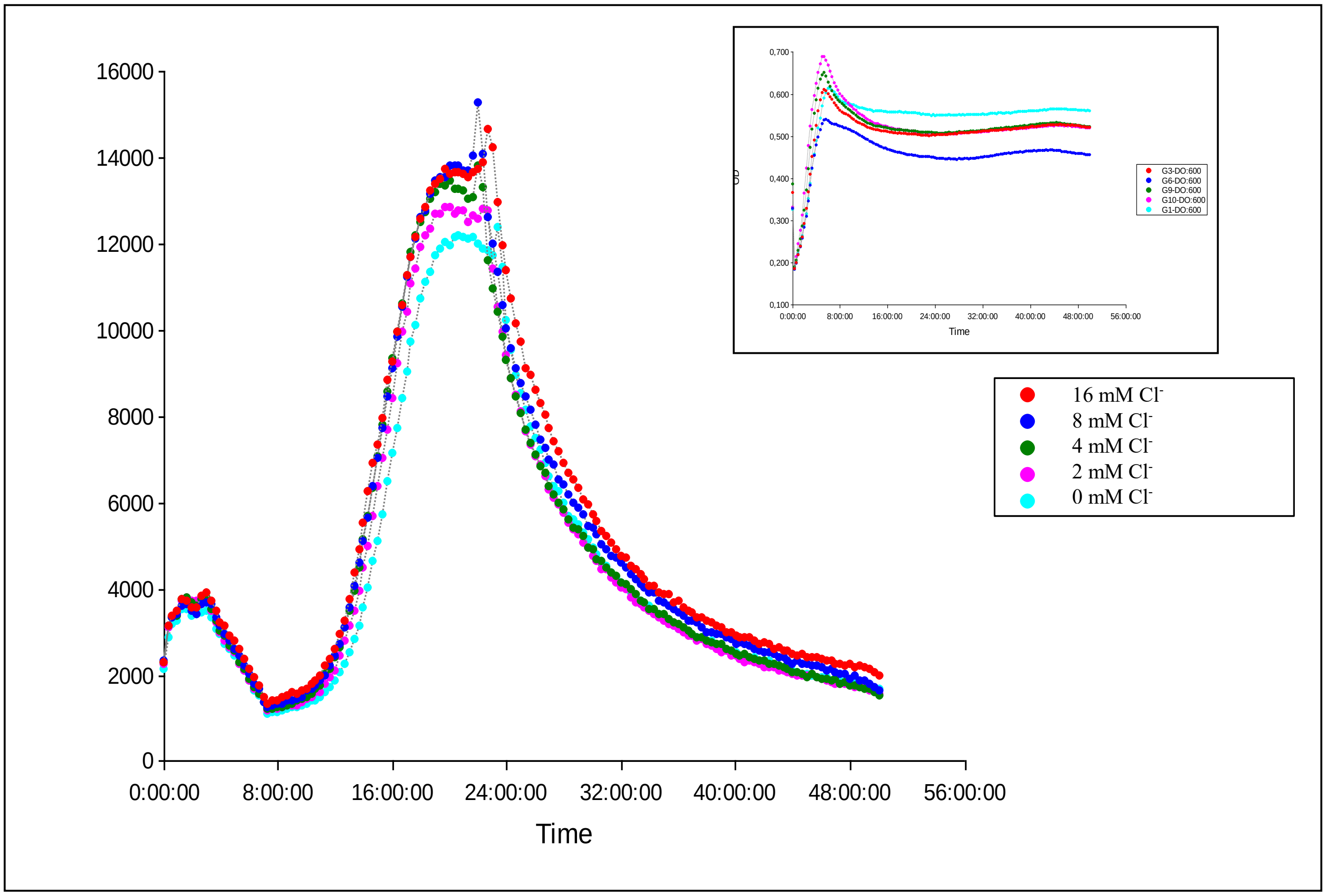

Figure S4. Testing chloride to look for potential osmolarity effects.

Bacteria with cloned reporters were cultured in presence of various concentrations of chloride to ensure that the observed effect could not be induced by other ions or general osmolarity effects. No major effect was observed on expression (luminescence, RLU), as pictured in figure above for *E. coli* SM10 λpir or on growth (inset). This is a representative assay picked from numerous assays performed with different combinations of fluoride and chloride concentrations and, while in some cases there appeared to be some Cl^- -mediated modulation (which we have not assessed as significant however), the fold induction from Cl^- was always less than 30% (typically less than 10%), much less than the fold induction from fluoride. We hypothesize, this might be due to some indirect effects with regards to how Cl^- may affect F^- availability, but we did not explore this further.

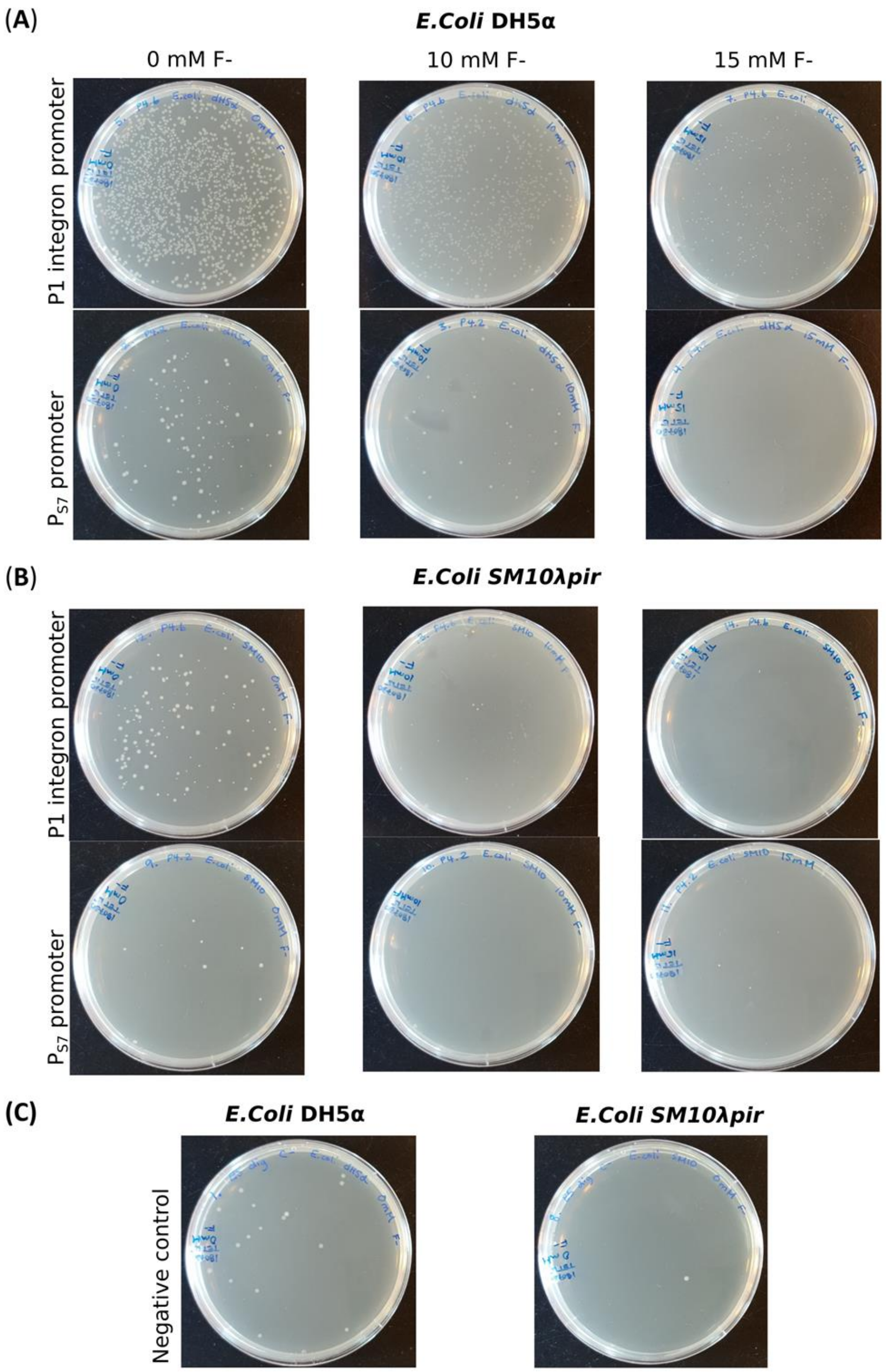

Figure S5: Transformation of heat-shocked *E. coli* is possible in high fluoride concentrations.

Transformation plates of GA reactions of the P1 integron promoter or the P$_{S7}$ promoter upstream of the *B. thailandensis* fluoride riboswitch controlled *lux* operon (**Table S1**: P1 integron promoter and P$_{S7}$ promoter, respectively), performed at different concentrations of fluoride into *E. coli* DH5α (**A**) or *E. coli* SM10λpir (**B**) after an overnight incubation at 37°C. Selection plates are of LB agar and are supplemented with 15 µg/mL tetracycline. Negative controls, of transformations with digested backbone only are also shown (**C**).

Table S4. P_{S7} promoter cloning sequencing results

Transformation fluoride conditions (mM)	Strain	Number of successfully sequenced clones	Number of clones containing 56 nt addition and C:T mutation in the P_{S7} promoter	Number of clones with a gap instead of the promoter sequence	Number of clones with an unrelated sequence instead of P_{S7}
0	*E. coli* SM10λpir	7	3	3	1
0	*E. coli* DH5α	9	2	2	5
10	*E. coli* DH5α	10	1	6	3
15	*E. coli* DH5α	4	4	0	0

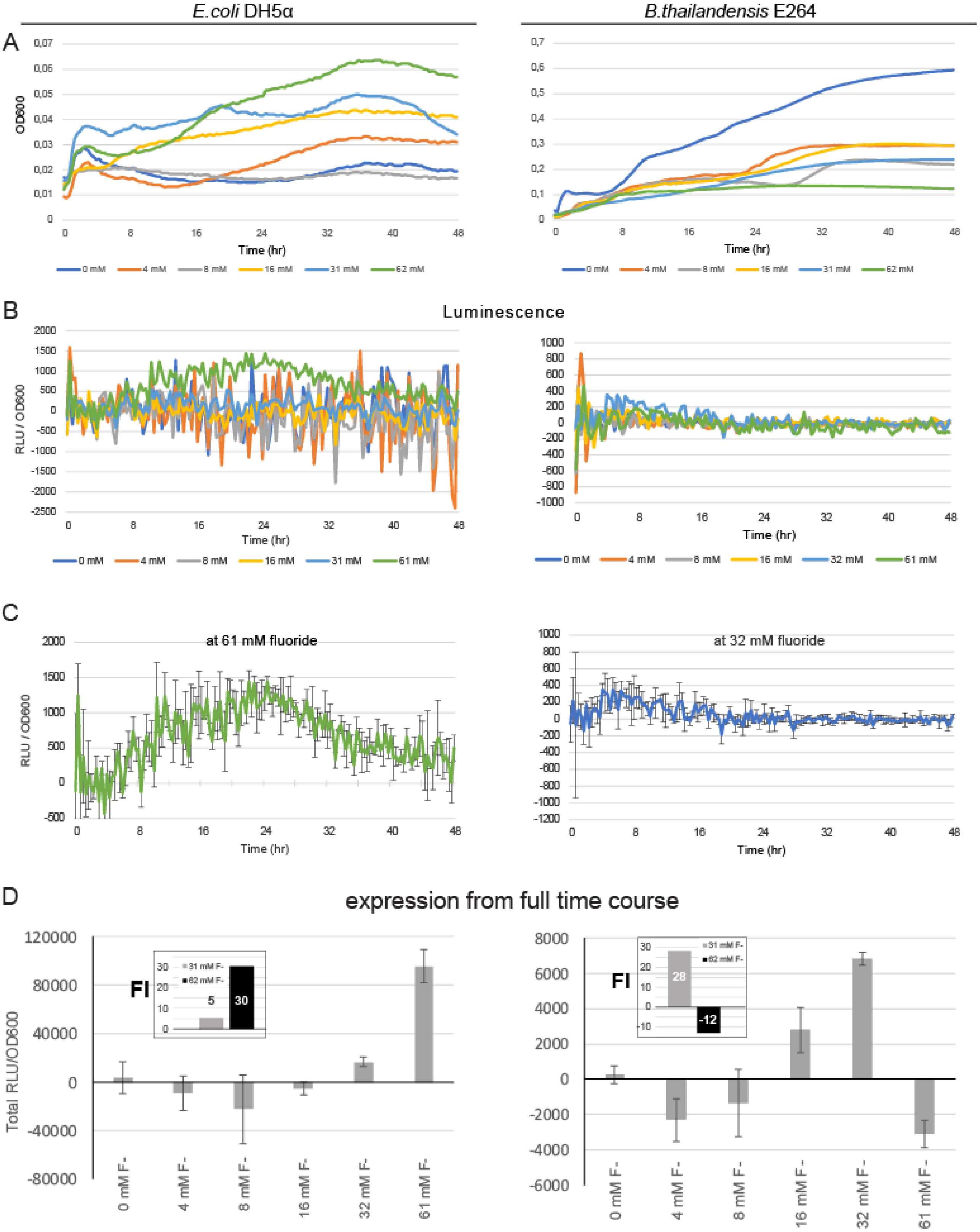

Figure S6. Double riboswitch constructs full time course.

(**A**) Growth curves of *E. coli* DH5α and *B. thailandensis* E264 cultures with varying concentrations of fluoride.

(**B**) and (**C**) *Lux*/OD over a 48h time-course regulation by the *Nitrosomonas europea* fluoride riboswitch with an expression platform (presumably) transcriptional cloned upstream of the *B. thailandensis* fluoride riboswitch used in the other constructs. Pre culture (LB) and expression media (0.5X MM) was supplemented with antibiotics as described in materials and methods.

(**D**) Total luciferase expression over the entire time course (from time 0 to 48h), calculated from triplicates for each concentration. Inset represents fold induction (FI).

While 61 mM fluoride does seem to induce expression (as much as 34 folds), the expression remains extremely low because it is strongly repressed by default and is compared to a value close to 0 luminescence, thus artificially inflating the FI. For *B. thailandensis* readings at 61 mM fluoride (as well as concentrations from 0 to 8 mM) the error bars that go into negative luminescence units illustrates how close we are to the time points, i.e. that many time points have a luminescence lower than that of the blank controls (media without bacteria).

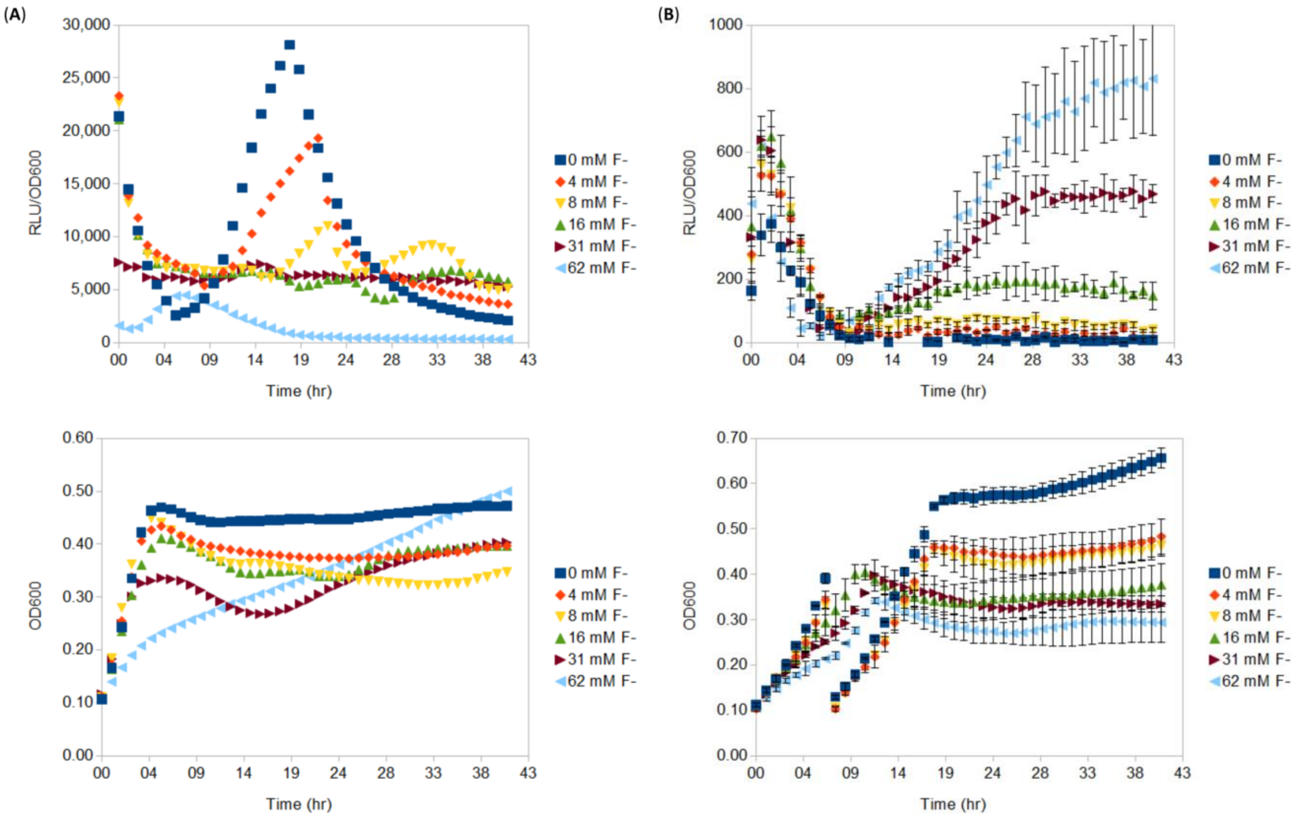

Figure S7: Regulation thresholds of the *B. thailandensis* fluoride riboswitch in *B. thailandensis* E264 and in *E. coli* SM10λpir.

(**A**) *Lux*/OD over a 42h time-course regulation by the *B. thailandensis* fluoride riboswitch in *E. coli* SM10λpir for a range of fluoride concentrations between 0 and 62.5mM. Pre culture (LB) and expression media (0.5X MM) was supplemented with tetracycline (15 µg/ml).

(**B**) *Lux*/OD over a 42h time-course regulation by the *B. thailandensis* fluoride riboswitch in *B. thailandensis* E264 for a range of fluoride concentrations between 0 and 62.5mM. Pre-culture (LB) and expression media (0.5X MM) was supplemented with tetracycline (25 µg/ml), gentamicin (50 µg/ml), and polymyxin (15 µg/ml).

Bottom graphs represent growth curves of strain cultures. The data points in a. represent single values while the data points in b. represent the means and standard deviations of triplicate values. All samples were measured on the same 96-well microplate run. Triplicate values for the *B. thailandensis* fluoride riboswitch construct in *E. coli* SM10λpir exist on other microplate runs and yield similar results (not shown).

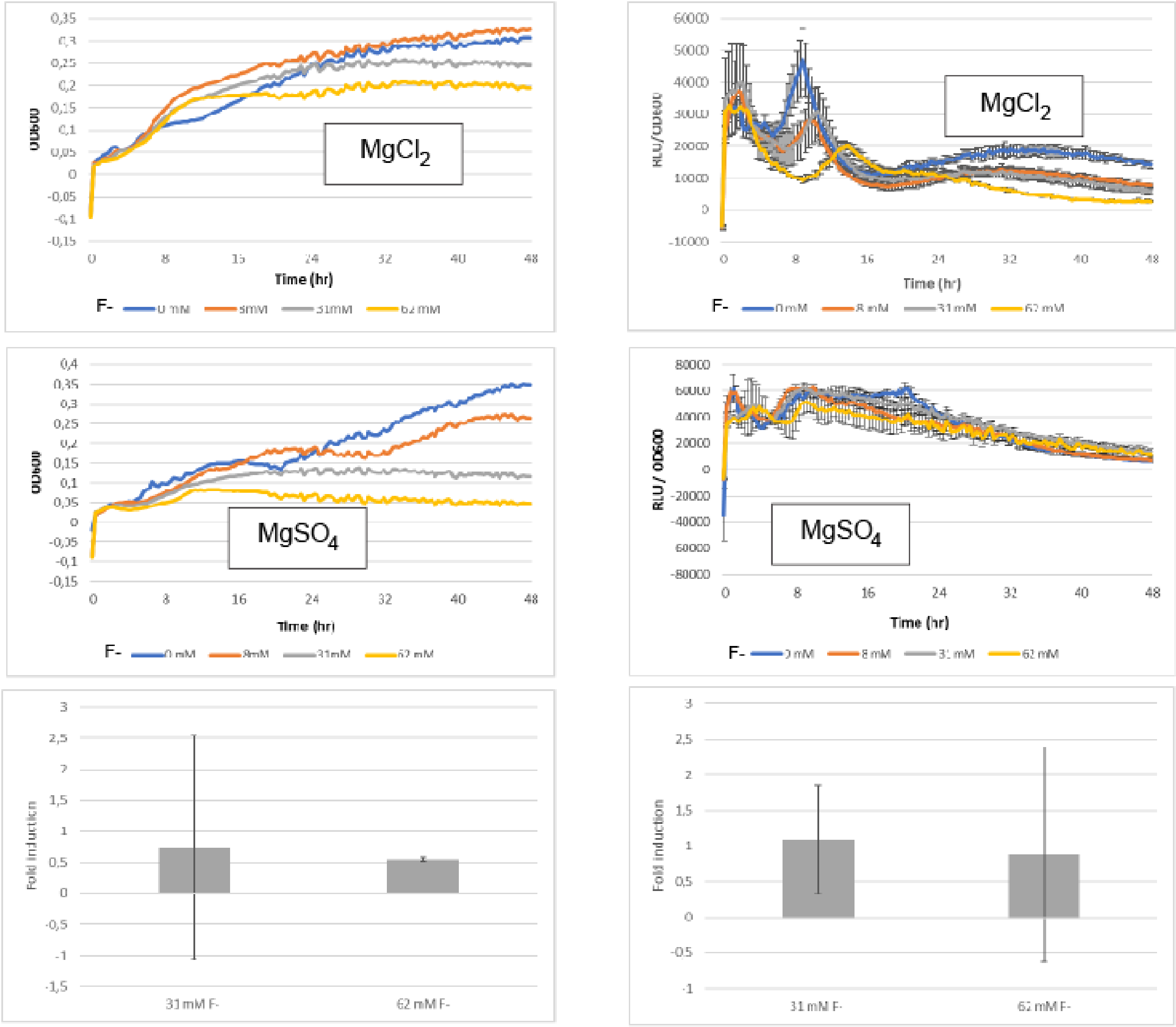

Figure S8: Fluoride has little effect on expression with the *metZ* 5ʹUTR in *B. thailandensis*.

We used the 5ʹUTR of the gene *metZ* (involved in methionine metabolism) as a negative control. As can be seen from the curves, whether in $MgCl_2$ or $MgSO_4$, no induction is observed with increasing concentrations of fluoride. Similarly, calculating fold induction (bottom) on the cumulative luminescence over the 48h of culture shows no significant change.

Table S5. pVK-f2-*lux* (P1 + *B. thai* F RS-*lux*) plasmid production and *lux* expression specifications in *E. coli* DH5α and *E. coli* SM10λpir.

Luciferase activity values and growth data were obtained from an 41h microplate incubation of 200 μL cultures containing the P1+*B. thai* F RS-*lux* construct (pVK-f2-*lux*) in presence of 3.91 mM fluoride, previously shown to be too low to induce riboswitch-mediated regulation. Values represent the means and standard deviations of triplicate results.

Strain	Plasmid production (total ng)[1]	Peak Lum (RLU)[2]	OD_{600} at Peak Lum	Peak Lum/OD_{600}	Average Lum	Average OD_{600}	Average Lum/OD_{600}	Fitness (OD_{600} Plateau value)
E. coli SM10λpir	4910 ± 175	10,400 ±100	0.31 ±0.01	33,700 ±400	5100 ±100	0.33 ±0.014	125 ±2	0.305 ±0.003
E. coli DH5α	6820 ± 403	2,120 ±90	0.39 ±0.03	5,400 ±300	1500 ±20	0.38 ±0.03	32 ±2	0.405 ±0.018

[1]Extracted from a 4.5 mL culture of 0.7 OD_{600}, details below in "Quantifying plasmid production in *E. coli* strains DH5α and SM10λpir"
[2]Both strains were tested on the same microplate during the same run.

Quantifying plasmid production in *E. coli* strains DH5*α* and SM10λ*pir*

20 mL of liquid Luria Broth supplemented with 15 µg/mL tetracycline was inoculated in a 50 mL falcon tube with *E. coli* DH5*α/*P1 *+ B. thai* F RS or *E. coli* SM10λpir*/*P1 *+ B. thai* F RS and incubated O/N at 37°C on a shaker. OD_{600} measurements were taken from each inoculation after vortexing and both cultures were diluted to an OD_{600} of 0.7 in a volume of 15 mL. 4.5 mL of each culture was used for plasmid extraction with the Genaid kit and plasmid concentrations were measured using a Nanodrop. Each extraction was repeated three times.

Potent promoter cloning bottleneck in *lux* hints at reporter gene toxicity

Cloning of the *B. thailandensis* E264 *metK* RBS sequence, 5'-AGGAGC-3', was attempted downstream of the S7 ribosomal protein gene promoter (P_{S7}) from *Paraburkholderia xenovorans* strain LB400 (recently renamed *Paraburkholderia xenovorans*), using two different 3-fragments Gibson Assembly design strategies, one of which had the P_{S7}+RBS fragment adapted for an XcmI/SwaI digested mini-CTX-*lux* backbone and the other which had the P_{S7}+RBS fragment adapted for a BsaAI/SwaI digested mini-CTX-*lux* backbone (**Table S1**). Vector assembly restriction enzyme digestion results suggested that 7 of the 9 obtained clones contained misassembled constructs (**Figure S1A and C**). The two vector assemblies containing correctly sized inserts were sequenced and revealed mutations which are suspected to have rendered clones non-luminescent.

A 56 nt sequence consisting of 5'-AACTGGGTTCGTGCGAGCTCATCGATTTCGTTCCACTGAGCGTCAGACCCCGTAGA-3', inserted between C13 and T14 of the P_{S7} promoter from pMLS7 (Lefebre and Valvano, 2002) and a point mutation of C285T of the promoter sequence was present in one of the clones, while the other clone contained a point mutation of G334T of the *luxC* sequence, modifying a glycine to a cysteine. Nucleotide BLAST analysis of the 56 nt insert against *Escherichia coli* K-12 revealed many possible origins (**Figure S1B**). We suspect this insertion and point mutation somehow blocked transcription from the P_{S7} promoter, or inactivated *luxC* as other clones obtained in experiments described in this paper also contained the same sequence characteristics (**Table S4**). It is important to note, that the P_{S7} sequence used for our cloning experiments is identical to the one annotated in pMLS7, however the beginning 25 bp of this sequence is absent from the originating host species *Paraburkholderia xenovorans* 28strain LB400, and thus the reoccurring insertion site in our experiments for the 56 additional nucleotides may not be present in the host species. Because only clones with mutations/insertions were obtained from the GA, we believe that the correct clones were not viable due to reporter gene toxicity.

Moreover, GA cloning into the same *lux* containing backbone was attempted for another potent promoter + RBS combination. The P1 integron promoter, previously shown to be an optimal potent constitutive promoter for single copy expression of fluorescent reporter genes in *Burkholderia* spp (Su et al., 2014) was amplified with the RBS sequence of 5'-AGGAGT-3' directly downstream with appropriate Gibson assembly overhangs which had been tested to work for other constructs (**Table S1: P1 + 5'-AGGAGT-3' RBS v1 and v2**). However, no clones containing the correct assembly were obtained. An attempt to clone P1 alone, which contains a potential RBS (5'-AGGATT-3') 11 nts upstream of *luxC* in-frame ATG, was also unsuccessful (**Table S1: P1 integron promoter—no additional RBS**).

The *B. thailandensis* E264 *metK* RBS sequence was successfully cloned into the lux backbone with the full original 5' non-transcribed region, containing the native *metK* promoter, as well as the 5'UTR (**Table S1: *metK* IGR— from *B. thai***). Equally 5'-AGGAGC-3' was successfully cloned into the *lux* backbone in combination with only the native 5' non transcribed region of *metK* (**Table S1: *B. thai metK* promoter + 5'-AGGAGC-3' RBS**).

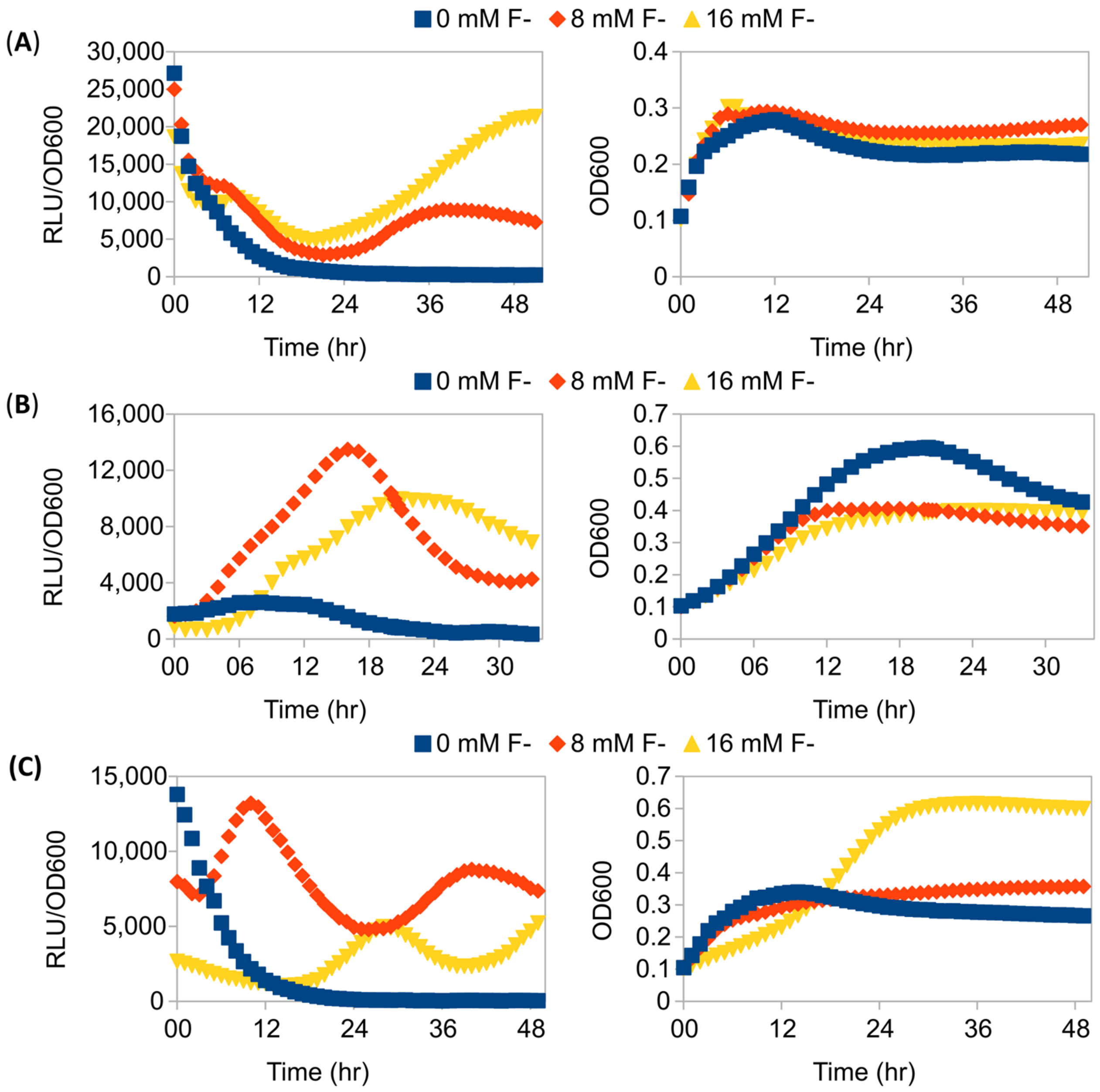

Figure S9: *P. syringae* fluoride riboswitch activity in *E. coli* DH5α reveals oscillating *lux* expression and variability across transformant strains.

RLU/OD_{600} (left) and growth curves (right) for a 52h, a 34h and 49h time-course expression assay of pVK-f-*lux* constructs in *E. coli* DH5*α*/P1 + *P. syr* F RS strain 1 (A), *E. coli* DH5*α*/P1 + *P. syr* F RS strain 2 (**B**), and in *E. coli* DH5*α*/P1 + *P. syr* F RS strain 3 (**C**) respectively. Luciferase expression portrays the variability of regulation across transformants. *Lux* expression is shown in presence of 0, 7.8 and 15.6 mM fluoride. Pre-culture (LB) and expression media (0.5X M9-MM) was supplemented with tetracycline (15 µg/ml). The data points represent the means and standard deviations of triplicate values measured on the same 96-well microplate run.

Quick User Manual for pVK-f-*lux*

(P1 + *P. syringae* fluoride riboswitch + *lux*)

Updated 2020-07-01

Vesta Korniakova

Intro: Why Use This Plasmid?

This plasmid is useful for down-regulating the *lux* operon through translation regulation via the fluoride riboswitch from *Pseudomonas syringae*. This is useful when you do not know why cloning/transformation or conjugation has been unsuccessful for your construct. A possible reason for your problem may be that the promoter which you are attempting to clone upstream of the *luxCDABE* cassette is too strong and is causing toxicity in your host bacteria, due to overexpression of lux. This modified version of the integration vector mini-CTX-*lux*, named pVK-f-*lux* contains an extra layer of regulation (the fluoride riboswitch) between the promoter and *luxCDABE*. In the absence of fluoride, *lux* is down-regulated by at least a factor of 2 in *Escherichia coli* DH5α. If maximal up-regulation is required, fluoride can be added to the growth media. In *E. coli* DH5α, fluoride concentrations between 7.8 and 15.6 mM, caused the strongest up-regulation effect while in *Burkholderia thailandensis* E264, the strongest effect was achieved with a 31 mM fluoride concentration. Growth curves at these concentrations, both in *E. coli* DH5α and in *B. thailandensis* E264 were moderately affected and should be monitored for individual experiments.

Plasmid Contents

This mini-CTX-*lux* modified plasmid contains:

1. P1 integron promoter flanked by 2 added multiple cloning sites (3 restriction sites on each side of promoter; MCS1: CsiI, XmaJI and SacI; MCS2: NcoI, SdaI and ScaI)

2. *P. syringae* fluoride riboswitch, flanked by 22 nt of upstream sequence and 271 nt of downstream sequence containing the beginning of the gene naturally downstream of the riboswitch (encoding a voltage-gated chloride channel family protein) in fusion with *luxC*, including a type IIS restriction enzyme site (AarI) between the two (without interrupting the fused ORFs).

3. *luxC* has an optimized codon for alanine for *B. thailandensis* E264, at position 7044…7046 nt. This was done to remove the AarI restriction site present in the original mini-CTX-*lux* plasmid, in order to relocate the AarI site upstream of *luxC*. Preliminary tests show no side effects of this mutation.

The key features and the pVK-f-*lux* map are presented in **Figure 1**. Additionally, the nucleic acid and amino acid sequence is shown in **Figure S2**.

Cloning Strategies

For swapping out the P1 integron promoter for a different promoter

Digest plasmid upstream of P1 integron promoter with either: XmaJI or SacI.

Digest plasmid downstream of P1 integron promoter with: NcoI, SdaI or ScaI.

For swapping the riboswitch out for a different RNA regulatory element:

Digest plasmid upstream of the fluoride ribowitch with: ScaI, SdaI or NcoI.

Digest plasmid downstream of the fluoride ribowitch with: AarI.

For swapping out the P1 integron promter and the fluoride riboswitch using one RE:

Digest plasmid upstream of the P1 integron promoter and downstream of the fluoride riboswitch with: CsiI. Note that with this option translational fusion is not possible.

*Important detail: AarI will cut after the 4th nt. of the original *luxC*, so make sure to add back 1 nt to re-establish the correct reading frame of *luxC*. Compare your design and make sure it contains the beginning of the original *luxC* amino acid sequence: M-T-K-K-I-S-F and ends with a stop codon. For translational fusion, the M start codon should be omitted however, to instead use the first codons of the coding sequence relevant to the regulatory element under study. This design was intentional as during Gibson Assembly cloning it allows screening for colonies containing plasmids that have re-ligated without the insert, as the exonuclease will chew back any 5' overhangs thus rendering original sequence re-ligation unlikely. The re-ligated plasmid colonies which do not contain the insert will have lost the required reading frame of *luxC* and will not be luminescent.

Useful tips

1. All of these enzymes use the same digestion buffer for promoter swapping: CsiI, XmaJI, SacI, NcoI and SdaI (10X FastDigest Buffer).

2. All of these enzymes use the same digestion buffer for RNA regulatory element swapping: ScaI and AarI (Fisher 10X Buffer AarI, AjiI, Bpu10I, PasI, ScaI).

3. If you want to mix and match different promoters and regulatory RNA elements (5'UTRs) for different modular constructs see below for tried-and-true Gibson Assembly (GA) overlaps.

Note that the underlined sequence is the recognition site of the restriction enzyme (RE) used corresponding either to the left or right cutter and bold sequence is not part of the GA overlap but is a recommended addition in order to re-introduce either the RE recognition site, the *luxC* original sequence, or both in the final construct. The *luxC* start codon is in bold and in italics. Spacing indicates the frame to match for proper *luxC* expression. If primer length is limiting the right GA overlap does not require the suggested re-insertion of the AarI RE site nor the beginning nucleotides of *luxC*. Do make sure to add 1 nt back instead of the latter in order to ensure the

correct reading frame of *luxC*. For translational fusions, it is preferable to delete the start codon to avoid the possibility that translation initiation might occur at this site rather than at the intended start codon more relevant for the system being studied.

a. Use left cutter SacI RE, and right cutter NcoI RE, for swapping promoters. The left and right GA overlaps to use, as they would appear in a 5′ to 3′ PCR-amplified insert fragment are shown on the left and right side of the insert sequence (N_n):
5′-ACCAGGTCCTAGG<u>GAGCT**C**</u>[N_n]**<u>CCATGG</u>**CCTGCAGGAGTACT-3′.

   If needed, a unique BsaAI RE recognition site is located further upstream of the P1 promoter and may also be used as a left cutter. For a BsaAI RE (outside the MCS1)-cut backbone, the left GA overlap to use is shown below:
5'-AAAGTTACAGGCCAGGAAC<u>CAC**GTA**</u>[N_n]-3'.

b. Use left cutter ScaI RE and right cutter AarI RE for swapping regulatory RNA elements. The left and right GA overlaps to use, as they would appear in a 5′ to 3′ PCR-amplified insert fragment are shown on the left and right side of the insert sequence (N_n):
5'- CCATGGCCTGCAGG<u>AGT**ACT**</u>[N_n]**<u>C ACC TGC</u>** ***ATG*** **ACT AA**A AAA ATT TCA TTC ATT ATT AAC GG-3'.

The above recommendations will allow to use the same GA insert design for all constructs, using the same GA overlaps instead of re-designing the overlap sequences necessary for each construct if cloning in a non-sequential manner.

4. Easy restriction digestion verification was envisioned with this plasmid. Cutting the P1 integron promoter out with SacI and NcoI will yield a 328 and a 49 bp band because there is a SacI site within the P1 promoter. Cutting out the fluoride riboswitch with ScaI and AarI will yield a 380 bp band. Bands (328 bp or 380 bp band), although faint, should be visible as long as 150 ng of digestion reaction is loaded into a small well (15-well comb for small 6x10cm agarose gel tray).

Examples of the above cloning strategies are represented in a flow chart for convenience.

Cloning flow chart

1. Cut the plasmid with the corresponding restriction enzymes.

To swap out the promoter, cut with SacI and NdoI
To swap out the ncRNA, cut with ScaI et AarI

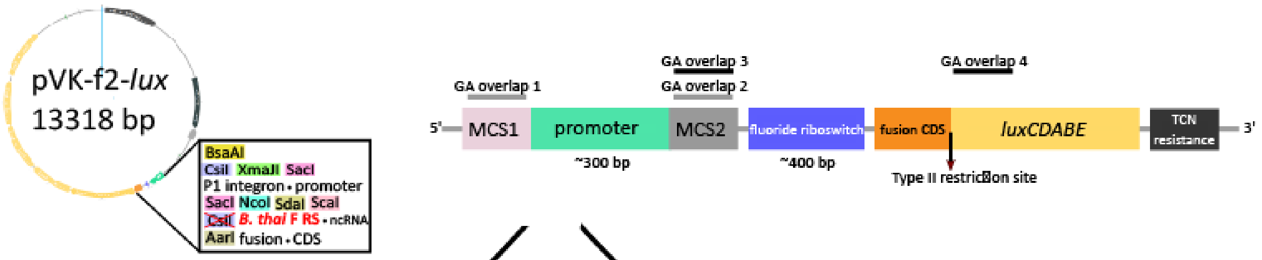

2. Using restriction enzymes to insert the fragment in the plasmid

2.1 Digest the fragment using the corresponding enzyme

To swap out, the promoter :

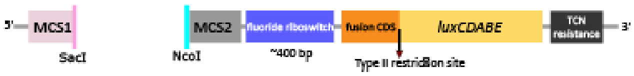

To swap out, the ncRNA :

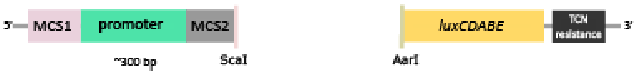

2.1 Link the fragment with the plasmid using T4 DNA ligase

2. Using Gibson Assembly to insert the fragment in the plasmid

To swap out, the promoter using the enzyme described above with the GA overlap 1 and 2:
5'-ACCAGGTCCTAGGGAGCTC[insert]CCATGGCCTGCAGGAGTACT-3'

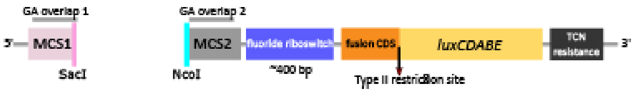

To swap out, the ncRNA using the enzyme described above with the GA overlap 3 and 4 :
5'-CCATGGCCTGCAGGAGTACT[insert]CACCTGCACTAAAAAAATTTCA TTCATTATTAACGG-3'

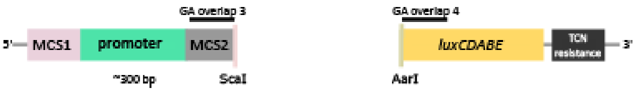

3. Transform in *E.coli* DH5α by thermal shock

4. Transform in *E.coli* sm10λpir by thermal shock

5. Conjugate in *B.thailandensis* E264

References

Becher, A., and Schweizer, H.P. (2000). Integration-proficient *Pseudomonas aeruginosa* vectors for isolation of single-copy chromosomal lacZ and lux gene fusions. *Biotechniques* 29**,** 948-950, 952.

Chen, J., Li, Y., Zhang, K., and Wang, H. (2018). Whole-Genome Sequence of Phage-Resistant Strain *Escherichia coli* DH5alpha. *Genome Announc* 6.

Simon, R., Priefer, U., and Pühler, A. (1983). A Broad Host Range Mobilization System for In Vivo Genetic Engineering: Transposon Mutagenesis in Gram Negative Bacteria. *Bio/Technology* 1, 784–791. doi:10.1038/nbt1183-784.

Kim, H.S., Schell, M.A., Yu, Y., Ulrich, R.L., Sarria, S.H., Nierman, W.C., and Deshazer, D. (2005). Bacterial genome adaptation to niches: divergence of the potential virulence genes in three *Burkholderia* species of different survival strategies. *BMC Genomics* 6**,** 174.

Lefebre, M.D., and Valvano, M.A. (2002). Construction and evaluation of plasmid vectors optimized for constitutive and regulated gene expression in *Burkholderia cepacia* complex isolates. *Appl Environ Microbiol* 68**,** 5956-5964.
